# Model linkage to assess forest disturbance impacts on water quality: A wildfire case study using LANDIS(II)-VELMA

**DOI:** 10.1016/j.envsoft.2024.106134

**Published:** 2024-09

**Authors:** Kar’retta Venable, John M. Johnston, Stephen D. LeDuc, Lourdes Prieto

**Affiliations:** a US Environmental Protection Agency (EPA), Office of Research and Development, Center for Environmental Measurement and Modeling, Ecosystem Processes Division, Landscape and Aquatic Systems Modeling Branch, 960 College Station Road, Athens, GA, 30605, USA; b US EPA Center for Public Health and Environmental Assessment, Office of Research and Development, Research Triangle Park, NC, 27711, USA

**Keywords:** Nutrients, Evapotranspiration, Precipitation, Forest management, Hayman fire, Water treatment

## Abstract

Wildfires in western US forests increased over the last two decades, resulting in elevated solid and nutrient loadings to streams, and occasionally threatening drinking water supplies. We demonstrated that a linked LANDIS (LANDscape DIsturbance and Succession)-VELMA (Visualizing Ecosystem Land Management Assessments) modeling approach can simulate wildland fire effects on water quality using the 2002 Colorado Hayman Fire. Utilizing LANDIS-II’s forest landscape model to simulate forest composition and VELMA’s eco-hydrologic model to simulate pre- and post-fire water quantity and quality, the best calibration performance yielded a Nash-Sutcliffe Efficiency (NSE) of 0.621 during 2000–2006 (most optimal annual - 0.921) in comparison to North American Land Data Assimilation System (NLDAS) runoff. Pre-fire modeled runoff, nitrate, and surface water temperature (SWT) correlated with observations. Simulated post-fire runoff (229%) and SWT (20.6%) were elevated relative to pre-fire, with nitrate concentrations 34 times greater than the aquatic life threshold (0.01 mg N/L).

## 1. Introduction

Due to climate change and human activity, there has been an increase in wildfire activity in many ecosystems beyond historic background levels. Wildfire activity in the western United States (US) forests has increased over the last two decades (Westerling, 2016), and fires have grown in quantity, scale, and strength across many ecosystems globally (Chow et al., 2021). Among many other effects, wildfires can impair downstream water quality by increasing post-fire runoff of sediment, nutrients, and organic material (Paul et al., 2022; Hohner et al., 2019; Rust et al., 2019; Bladon et al., 2008; Smith et al., 2011). These inputs in turn lead to challenges in municipal drinking water treatment, including increasing the levels of solids and the potential formation of harmful disinfection byproducts (DBPs) (Hohner et al.; Pennino et al., 2022). Changes in fire regimes (e.g., increases in fire frequency or severity) may fundamentally alter ecosystem structure and composition, such as shifting tree-dominated ecosystems, complicating future management responses and modifying inherit risk calculations.

Because of variation in watershed and fire characteristics and the need to evaluate various management interventions (e.g., thinning, prescribed fire, riparian buffer zone management, or riparian plantings), it is beneficial to have a modeling platform to explore disturbance events and management scenarios to understand water quality outcomes relevant to water treatment and water quality standards. The first priority in our research is to determine if a linked LANDIS-VELMA model can be achieved. The next objective is to better understand the multi-media effects of fire disturbance in relationship to biomass coverage, species, and age classification. We emphasized on water quality, quantity, and water budget parameters which can be validated from observed and modeled data before and after the fire. This is extremely relevant in regions where management scenarios are not performed or historically practiced, have sparse fire severity history, and limited timeseries of continuous observed validation data pertaining to water quality and quantity. Catchment ecohydrological dynamics are influenced by spatial and temporal climate patterns, land use and management practices, and the distribution of biomass (Dlamini, 2010) within the watershed. Customization of local wildland fire mitigation strategies may be necessary to maintain water quantity and quality during and after the disturbance. Terrestrial vegetation disturbance induced by wildfires has pronounced impacts on regional water cycle (water budget) behavior including reductions in infiltration, ground water recharge, and evapotranspiration rates with increases in surface runoff (Rodrigues et al., 2019; Moody and Martin, 2009; Smith et al., 2011). Currently, models individually lack the capability to fully evaluate tradeoffs across ecohydrology, biogeochemistry, and management practices simultaneously. Basso et al. (2022) explains current models are limited capturing small time scale fate, transport, and long-term impacts associated with post-fire water quality which can reoccur during extreme precipitation events consequently increasing residence times in reservoirs controlled by watershed and ecosystem dynamics (Emelko et al., 2015).

Most wildfire modeling research is focused on simulated fire scenarios based on burn severity rather than the validation of historical fires with the use of pre- and post-fire observations (Basso et al., 2022; Feikema et al., 2011; Morrison and Kolden, 2015; Basso et al., 2021). Current models utilized for wildfire and biomass disturbance include the Soil & Water Assessment Tool (SWAT) (Arnold et al., 1998), E2, Erosion Risk Management Tool (ERMIT) (Robichaud et al., 2014), Revised Universal Soil Loss Equation (RUSLE) (Renard et al., 1997), Normalized Burn Ratio (NBR) (Dwyer et al., 2018), Noah-Multiparameterization Land Surface Model (Noah-MP LSM) (He et al., 2023), Weather Research Forecasting System and SFIRE (WRF-SFIRE) (Mandel et al., 2011), Blue Sky (Larkin et al., 2009), and LandTrendr (Hooper and Kennedy, 2018) are mostly focused on land use classifications, burn fuel loads, smoke dispersion or soil, and limited in simplicity of parameterization and temporal resolution. Only a limited number of models quantify post-fire water quality predictions and utilize detailed vegetation classification. Each model separately has its own strengths in tackling issues surrounding the wildland urban interface (WUI).

**Fig. 1.**
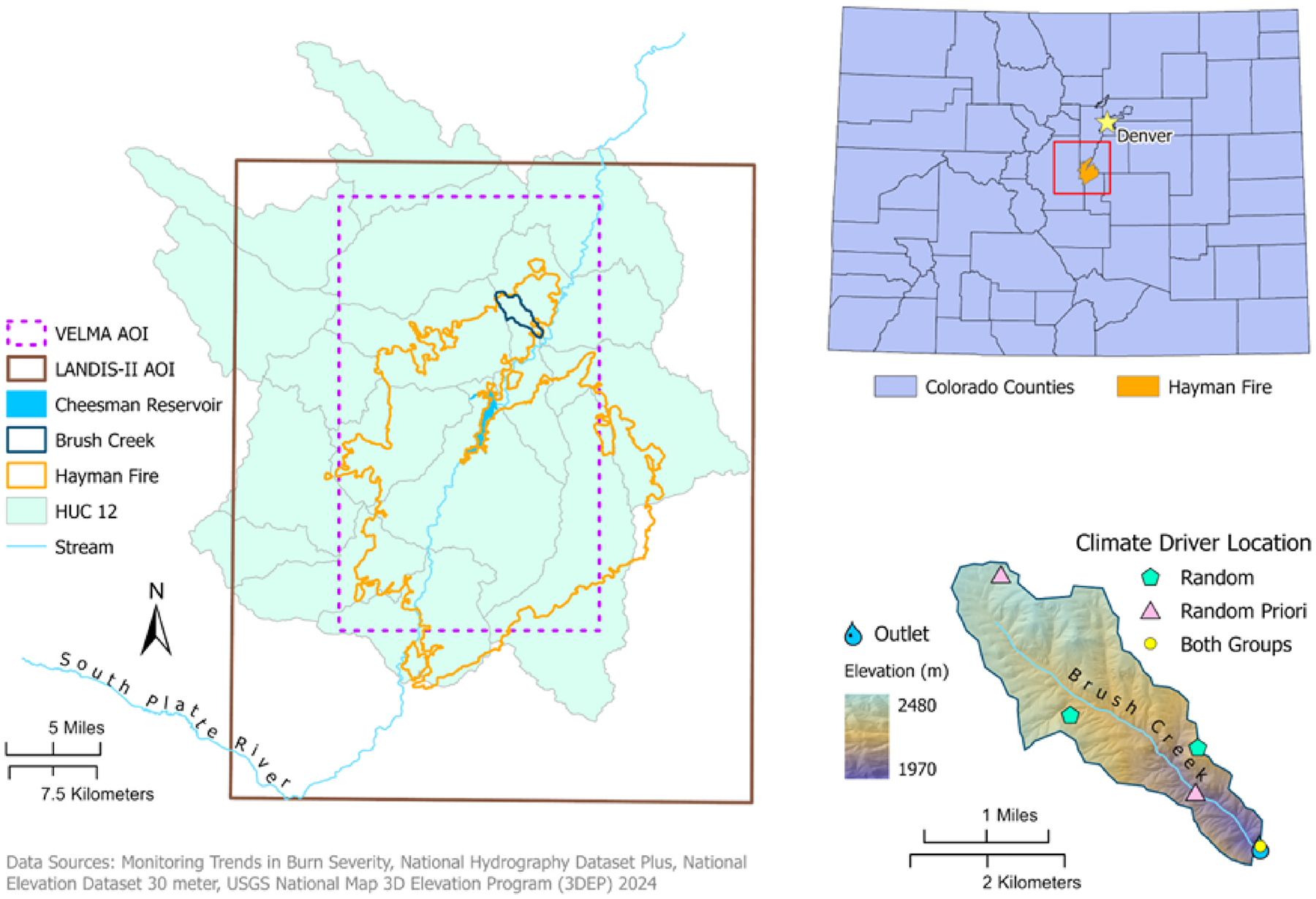
Location map showing the LANDIS-II and VELMA study areas, the 2002 Colorado Hayman Fire perimeter, the12-digit hydrologic unit code (HUC) boundaries, the climate driver locations used during simulations, and the VELMA delineated Brush Creek watershed with its outlet.

Because no current model encompasses all ecosystem processes necessary for fire science applications, we sought to link the dynamic landscape/disturbance model LANDscape Disturbance and Succession (LANDIS-II) with the ecohydrology model Visualizing Ecosystem Land Management Assessments (VELMA). Grid-based ecohydrology and biogeochemistry models, such as VELMA (US EPA, 2022) and LANDIS-II (Scheller et al., 2007), simulate the effects of environmental stressors, nutrient dynamics, ecological succession, and landscape disturbances. Here, we implemented and tested a linked LANDIS-VELMA model using the Hayman Fire of 2002 in Colorado as a case study (Fig. 1).

This paper examined calibrated model performance, compared the water budget to literature values and modeled data, and utilized measured streamflow and water quality parameters closest to the outlet delineation. Using this modeling platform, we specifically investigated: 1) the response of streamflow and nutrient loading before and after fire disturbance; and 2) the potential water quality effects of these endpoints prompting responses necessary for mitigation and management practices.

## 2. Study area and modeling framework overview

### 2.1. Case study description

To test the linked model workflow, we utilized a forested study area in the Front Range of the southern Rocky Mountains in Colorado. The spatial extent of the LANDIS-II study boundary was approximately 2430 km^2^ and contained a VELMA rectangular area of 829 km^2^ (Fig. 1). We simulated the 2002 Hayman Fire, one of the largest wildfires recorded in Colorado history (Wright, 2021). It occurred from June 8 through July 18, 2002 (McHugh and Gleason, 2003) burning roughly 138,114 acres) (McHugh and Finney, 2003) across four counties southwest of Denver: Jefferson, Teller, Park, and Douglas (Finney et al., 2003). In its path was the Cheesman Reservoir, a source of drinking water for Denver and surrounding communities in the South Platte River Basin (Denver Water, 2021). Ultimately, the Cheesman Reservoir intake was closed five years due to increased sediment post-fire. The delineated drainage basin.

(39.353°, −105.172°) within the area of interest (AOI) is 567 km^2^, comprises several hydrologic unit code (HUC) 12 catchments within the 10190002 8-digit HUC watershed (Fig. 1). For the initial linked LANDIS-VELMA calibration, we placed emphasis on the northern tier of the study area, the Pine Creek-South Platte River catchment. Due to limited availability of pre- and post-fire water quality and quantity data throughout the study area and the burn severity of the catchment, Brush Creek (39.263°, −105.227°) reach was representative of the study area for calibration. Model AOIs were overlapping, with the LANDIS-II boundary larger than VELMA (and VELMA AOI larger than the study area for ease in future extrapolation of other catchments).

### 2.2. Modeling framework

To initialize the linked modeling platform, several inputs were obtained to best represent the physical environment for both VELMA and LANDIS-II (Table 1). LANDIS-II is a gridded, stochastic, spatially-dynamic forest landscape simulation model that has been applied throughout the world (The LANDIS-II Foundation (TLF), 2022b). It simulates spatiotemporal biomass dynamics through growth and succession incorporating the life history attributes of trees and shrubs. Best suited for modeling areas greater than 10,000 ha (TLF, 2018a), LANDIS-II uses separate modules, called extensions, to simulate a variety of landscape disturbances (e.g., fires, wind events, harvesting, insect outbreaks) over time spans of decades or centuries (TLF, 2022a). VELMA is a gridded, ecohydrological model that utilizes land surface hydrology and terrestrial biogeochemistry (plant-soil) to simulate the integrated responses of vegetation, soil, and water resources to changes in climate, land use, forestry management practices, and disturbance (McKane et al., 2014). All required input data were masked to each model’s AOI boundary using a 30-m grid in the North American Datum (NAD) 1983 Universal Transverse Mercator (UTM) Zone 13N projection (Fig. 1).

In the VELMA AOI (Fig. 1), 85% of the LANDIS-II derived biomass, utilized in the linkage, was dominated by coniferous evergreen species (Rocky Mountain (RM) Douglas-fir, RM ponderosa pine, Engelmann spruce, and RM lodgepole pines). Sloping soils in the region are derived from weathered Pike’s Peak Granite batholiths and other metamorphic rocks and are categorized as fine sandy loam or loam with thin O-horizons (United States Department of Agriculture (USDA), Natural Resources, 2016; Retzer, 1949; Cipra et al., 2003; Abella et al., 2013). Topographical driven climatic differences occur in the Lower and Upper Montane areas, associated with a moisture gradient between sheltered slopes (wet) and exposed ridges (dry) (Addington et al., 2018). In addition to the highly variable terrain, explicit differences in solar radiation, precipitation, and latitudinal gradients exist, consequently impacting (Addington et al.; Veblen et al., 2000) soil moisture, evapotranspiration rates, and streamflow, driven by a divisional line separating the Front Range denoted as the Palmar Divide. The case study AOI (Fig. 1) is situated nearly in the middle of this divisional line. This creates spatial-temporal heterogeneity with precipitation varying from the spring to summer between the northern and southern mountains, increasing the complexity of accurately modeling this region.

### 2.3. VELMA biomass & model linkage

We utilized the age and biomass characteristics of the forest communities derived from LANDIS-II as described below to initialize and link with VELMA for pre-fire, post-fire, and high wildfire severity conditions. We simulated the Hayman Fire by incorporating VELMA’s leaf-root-stem (LRS) harvest disturbance. The disturbance was set to occur on June 10, 2002, reflective of substantial fire spread after initialization, using the USGS & USFS 2014 MTBS fire boundary polygon as a mask. This mask is used as a spatial filter determining which cells are impacted by the disturbance. The initialization concentrations of each cell in the AOI were developed from total aboveground (AG) biomass fractions and spatial pools measured and calibrated for HJA VELMA simulations (Table 2, McKane et al., 2014) merged with LANDIS-II AG communities characteristics.

Parameterization of the 95% biomass mortality was simulated through a LRS disturbance filter incorporating a 10% AG biomass offsite removal (Table 2) to mimic a wildfire using different climate driver locations, R and RP, and altering the LANDIS biomass median age and AG amounts assessing pre-fire (*) and post-fire simulation branches. The 6 orders of magnitude lower biomass in RA1L* depicts a high severity burn which quickly removes most of the fuel loads barely impacting water quality. We effectively calibrated the models through adjusting the vertical and lateral surface saturated hydraulic conductivity exponential decay factors impacting infiltration rates.

## 3. Calibration, metadata, methods, & analysis

### 3.1. LANDIS-II calibration

To provide VELMA with aboveground biomass, forest community species, and ages representative of pre- and post-fire conditions, we used LANDIS-II version 7.0 (TLF, 2018b) paired with the Biomass Succession extension (version 6.0) (Scheller and Mladenoff, 2004), the Biomass Output extension (version 3.0) (Scheller and Mladenoff), and the Age Cohort Statistics extension (version 3.0.1) (Ward and Scheller, 2018). LANDIS-II required a series of inputs (Table 1) including a raster of ecoregions or land types (TLF, 2018c) and an initial forest communities raster. We developed the ecoregions for our study area based on elevation, slope, temperature, and precipitation. These regions (Appendix A Fig. 1) roughly corresponded to the foothills, montane, subalpine, and alpine Colorado life zones (Addington et al., 2018; Kaufmann et al., 2006; Ramaley, 1907).

**Fig. 2.**
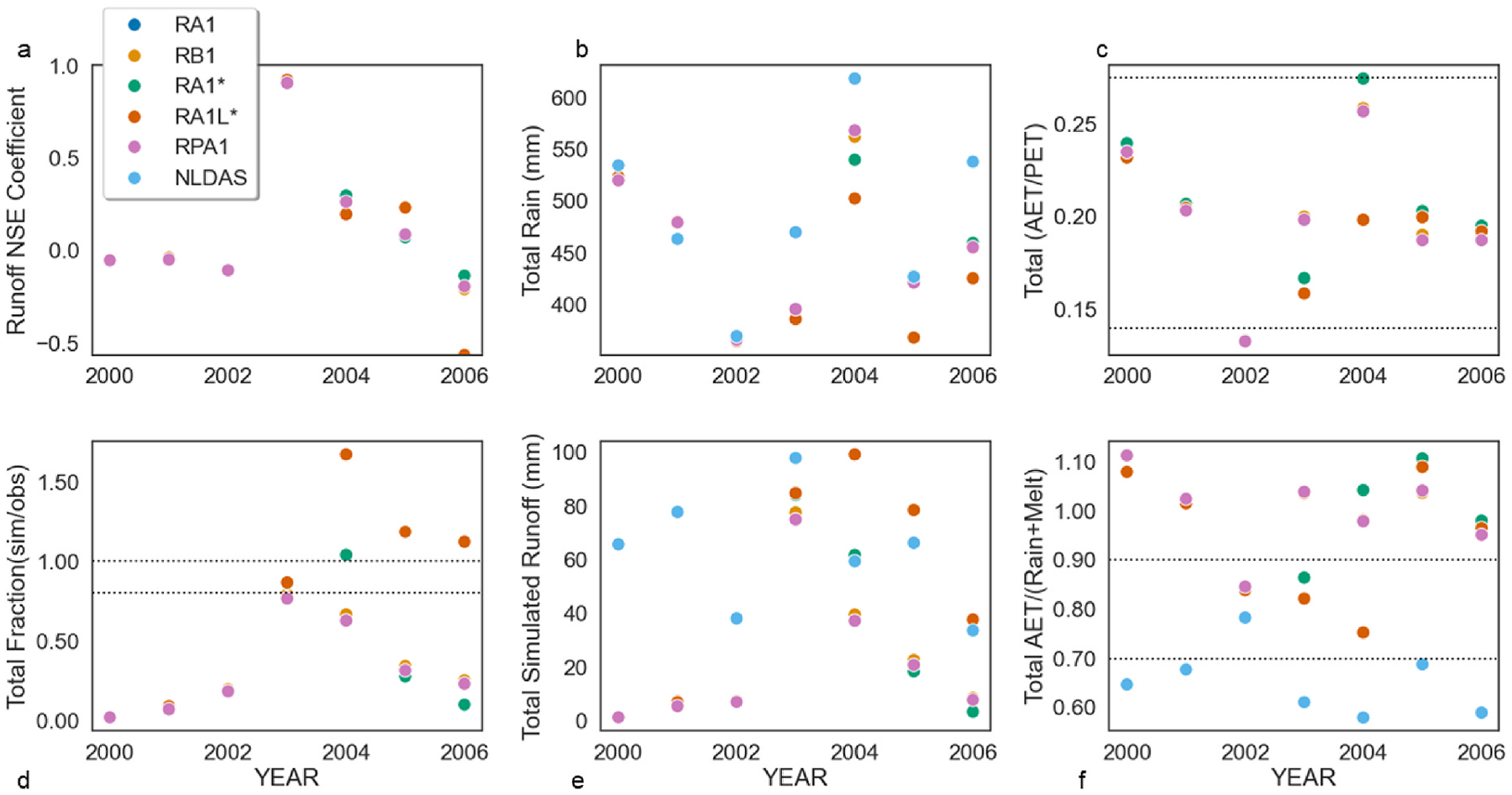
Evaluation of LANDIS-VELMA performance for annual water budget parameters scatter plots. (Top – *From Left to Right)* (a) Simulation performance comparisons of annual NSE; (b) Simulated rainfall in comparison to observed NLDAS (c) Simulated Actual Evaporation (AET) and Potential Evapotranspiration (PET) ratio compared to Peng et al.’s (2019) interquartile range. (Bottom – *From Left to Right*): (d) Simulated annual runoff comparisons with NLDAS relative to optimal performance (Moriasi et al., 2007; Waseem et al., 2017); (e) Simulated annual runoff; (f) Simulated AET/(Rainfall + snowmelt) compared to Sanford and Selinck (2013) and Daly et al. (2008). Note: Similar simulated results pertaining to their water budget may have overlapped markers.

In the initial communities raster, trees were grouped by species or functional groups and age cohorts (TLF, 2018a). We targeted the species composition and aboveground biomass of the forest communities in the region around the year 2000. Iterative adjustments were made to LANDIS-II simulated total aboveground live biomass until the value was within 1% of the biomass estimate derived from the National Biomass and Carbon Dataset (NBCD, 2000) (Kellndorfer et al., 2009, 2013) (Appendix A Fig. 2).

To simulate the 2002 Colorado Hayman Fire, we used LANDIS-II Dynamic Fire System and Dynamic Fuel System extensions (version 3.0) (Sturtevant et al., 2009) coupled with a fire ecoregions raster that constrained the burning to the inside of the Hayman perimeter (Appendix B Fig. 1). The extensions’ parameters were adjusted until the total aboveground live biomass inside the Hayman fire perimeter was within 1% of the biomass estimate derived from the year 2002 band of the LandTrendr Biomass, CONUS (1990–2017) dataset (Hooper and Kennedy, 2018) (Appendix B Fig. 2).

### 3.2. VELMA calibration

#### 3.2.1. Nutrient dynamics

This linkage was further performed using the LANDIS-II biomass arguments within a Python script to develop the average of 59 gridded AOI biogeochemical carbon and nitrogen initialization balances partitioned between the LRS and detritus spatially and temporally above and below ground (McKane et al., 2014, Table 1) which regulate biomass succession rates. Parameterization for each soil type was established based upon the US General Soil Map (STATSGO2) (USDA Natural Resources, 2016), literature review and soil surveys of the Front Range (Price and Amen, 1984; Meinke, 2004, Tables 1 and 3). Total soil profile depth was set to 1651 mm to reduce computational burden, with a pH of 6.5 utilizing a four-layered soil structure simulating mass balance flow contributions from each layer (Golden et al., 2014). Data obtained from STATSGO2 (USDA Natural Resources) database and McKane et al. (2022) were used to develop the four soil layers (Table 3). Porosity, field capacity, wilt point, and bulk density were calibrated for each soil type (McKane et al., 2014) (Table 2). VELMA soil and hydrologic parameters were modified from the HJA calibration. Calibrations utilized an overall hydraulic conductivity of 18000 mm per day with vertical exponential decay factor at 0.009 and lateral decay factor at 0.007. Ultimately, we used adjusted infiltration rates with the lateral decay factors, *K*_*sl*_, in the water, sandy loam, and loam soils using values of 0.00325 (^A^) and 0.00310 (^B^) representative of higher and lower infiltration rates within the soil (Table 2, Table 3 (A – Larger Infiltration Rates; B – Lower Infiltration Rates)).

#### 3.2.2. Watershed dynamics

Using the Hayman Fire boundary, we obtained NASA JPL SRTM V3 DEM (Farr et al., 2007, Table 1) and VELMA AOI polygon boundary from GEE. The resulting DEM watershed was delineated using the experimental enhanced flat processing algorithm in JPDEM (Pan et al., 2012, Table 1). To validate the delineated subwatershed stream network and pour points, the DEM value was checked against cell index values from the corresponding NHDPlus V2 COMID (US EPA & USGS, 2012, Table 1). To drive watershed climate dynamics, we utilized VELMA’s multiple weighted location spatial weather model to simulate daily temperature and precipitation based on three NLDAS latitude and longitude points including the outlet. The other two locations were randomly (R) selected and against an elevational random priori (RP) sampling within the catchment boundaries, which correlated with the point’s daily temperature and precipitation from 1992 to 2019 acquired from the US EPA HMS (2020–2021; Table 1). The parent, the worst performing, simulation, relies on the default single station weather model with data retrieved at the outlet point.

#### 3.2.3. Model comparison of simulated to observed results

To validate VELMA water quantity and quality outcomes, we compared simulated to empirically modeled results, literature values, observed streamflow, and observed water quality measurements annually, monthly, and pre- and post-fire. VELMA daily simulated runoff was compared to NLDAS daily surface runoff for each corresponding NHDPlus V2 COMID (retrieved from the HMS REST API, Table 1) between 1998 and 2006. Since a two-year spin-up is required, model performance evaluation is assessed during 2000–2006. VELMA model performance was evaluated using the Nash-Sutcliffe Efficiency (NSE) coefficients (Nash and Sutcliffe, 1970; Waseem et al., 2017), calculated against modeled data from NLDAS. To compare observed streamflow to VELMA and NLDAS daily runoff, observed daily discharge conversions were performed using a lambda scaling function and Scipy’s (Virtanen et al., 2020) 1-D interpolation library applying a nearest argument in Python using 2344 nodes. Data were resampled for 10, 30, and 120-day mean time series analysis for the simulated and observed data. Additional comparisons were performed to monitored streamflow data and water quality measurements, available from August 2001 through 2006 (disturbance interval) with approximately one monthly observation at Brush Creek (D. Pierson and T. Fegel, personal communication, January 11, 2021). Descriptive statistics, analysis of variance, and percentage change calculations were performed for data comparisons and analysis of modeled and observed results.

To quantify pre- and post-wildfire water quality and quantity impacts, we analyzed VELMA daily simulated runoff, stream surface water temperature (SWT), dissolved organic nitrogen (DON), dissolved organic carbon (DOC (measured as C)), ammonium (NH_4_), nitrate (NO_3_) during the disturbance interval. Monitored water quality parameters assessed (D. Pierson and T. Fegel, personal communication, January 11, 2021) were similar to VELMA loss outputs, excluding pH. Further analysis was performed on the VELMA simulated water budget parameters including daily averaged precipitation and evapotranspiration in comparison to modeled NLDAS values. Water quality exceedances were tracked (Table 4) and compared to several sources including the US EPA Stressor Identification Guidance (2000b).

These parameters were compared to catchment delineated average concentrations at the stream outlet during the disturbance interval against any of the exceedance criteria thresholds (Table 4). Because ammonia (NH_3_) concentrations are conditionally filtered by pH, additional pH computation (Stumm and Morgan, 1996; Park and Clough, 2018) was required utilizing VELMA DOC loss daily fluxes and surface water temperature. Estimates of dissolved carbon dioxide (CO_2 [aq]_) were derived from DOC loss scaled by 4, representative of the 4 M equal chemical compounds for the dissolution of the DOC (Stumm & Morgan) using pre-fire mean alkalinity observed (Table 4) for pH calculations. Ranges of pH were restricted to 3.5 to 8.5 according to the US EPA’s AQUATOX (Park & Clough) guidance. Conversion of delineated averaged nutrients from VELMA were converted to concentrations established by scaling the loss flux by the bankfull depth of the COMID (0.35 m), as stated in the NHDPlusV2.1 Bankfull Hydraulic Geometry (Wieczorek et al., 2018). NH_4_ transformations to NH_3_ were based upon Hach methodology which uses a scaling factor of the molecular masses to derive the cumulative estimate of Total Ammonia as Nitrogen (TAN). The following results represent the 5 best LRS wildfire simulations (Table 2) of the LANDIS-VELMA linkage.

## 4. Results

### 4.1. Overall water budget

Linked modeled (Table 2, A1–3, B1–3) predicted streamflow compared well to Brush Creek NLDAS streamflow. Between 2000 and 2006, after an initial two-year spin up, overall NSEs ranged from 0.601 to 0.621 (Table 2) displaying good fit, according to Moriasi et al. (2007) and Waseem et al. (2017). Annual optimal performance, or very good classification according to Moriasi et al. and Waseem et al. occurred in 2003 where predictive skill ranged between 0.905 and 0.921 for all simulations (mean 0.917) (Fig. 2a).

Maximum NSE of 0.921 was observed in RA1* (Fig. 2a and d) during high flow events resulting in a modeled runoff difference of 13.7 mm. The lowest modeled difference of 2.4 mm also occurred in the RA1* during 2004. Annually, NSE coefficients perform as well as the means increasing post-fire (Fig. 2a). Utilizing the spatial weather model resulted in an increased predictive skill of the model over 50.4% (Table 2) in the subwatershed.

Total rain plus snowmelt (Fig. 2b) observations agreed with Sanford and Selnick (2013) and Daly et al. (2008). Approximately 79% of simulated Actual Evapotranspiration (AET) values agreed with Sanford & Selnick’s regional estimation of ET which ranged from 310 to 500 mm. The majority of VELMA’s annually averaged AET to potential ET (PET) fractions matched the interquartile range for observations classified as evergreen needleleaf forest (ENF) (Fig. 2c), according to Peng et al. (2019). ET estimates of RA1 run exhibited greater covariance than other simulations and displayed statistical significant differences between NLDAS and RA1 performing an analysis of variance (ANOVA) (F = 165, p = 3.80 E–37). RA1L* daily averaged ET simulations only differed 26.3% from NLDAS. ANOVA between VELMA (RA1, RA1* and RA1L*, RB1, RPA1) and NLDAS daily average AET demonstrated significant differences (F = 47.6, p = 4.63E-49). The total simulated annual runoff comparisons were most comparable with NLDAS during 2003–2006 (Fig. 2e). Almost 40% of total AET and rain plus snowmelt were within the estimated precipitation lost to ET during 1971–2000 according to Sanford & Selnick. VELMA modeled estimated mean annual actual evapotranspiration (AET) and rainfall plus melt fractions performed most optimal during post-fire in simulations (Fig. 2f).

**Fig. 3.**
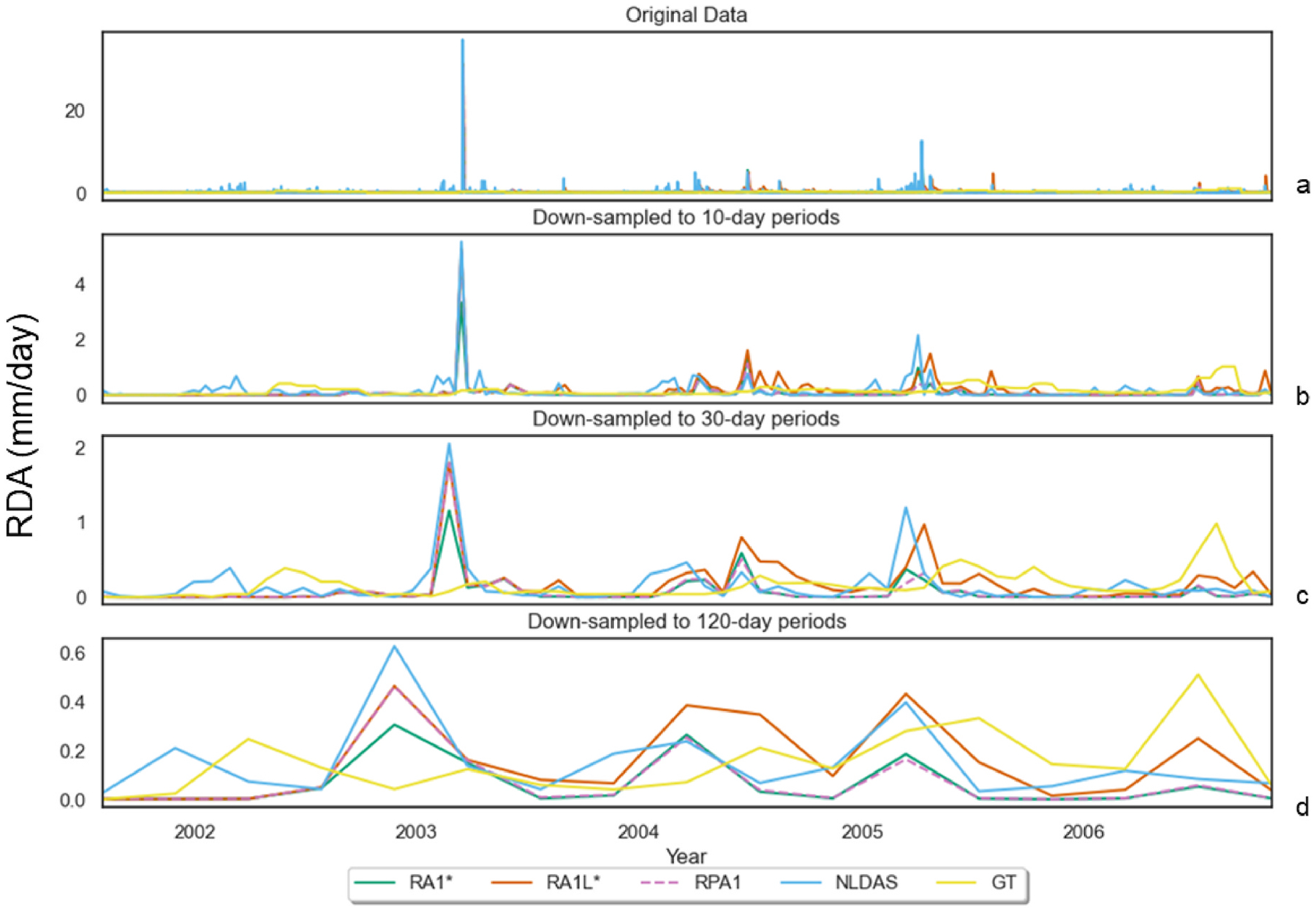
Time series of resampled mean runoff delineated averages (RDA) for all simulations (shown: RA1*, RA1L*, & RPA1) and NLDAS in comparison to limited nearest neighbor 1-d interpolation of observed runoff (a) for 10 (b), 30 (c), and (d) 120-day sample periods during the disturbance interval. Best performance occurs in RA1L* & RA1*using LANDIS pre-fire AG conditions, especially during low flow events in comparison to observed runoff. Differences exist in the y-axis due to reductions in local maximums during resampling which allow the best view of the timeseries trends for each resampled period.

**Fig. 4.**
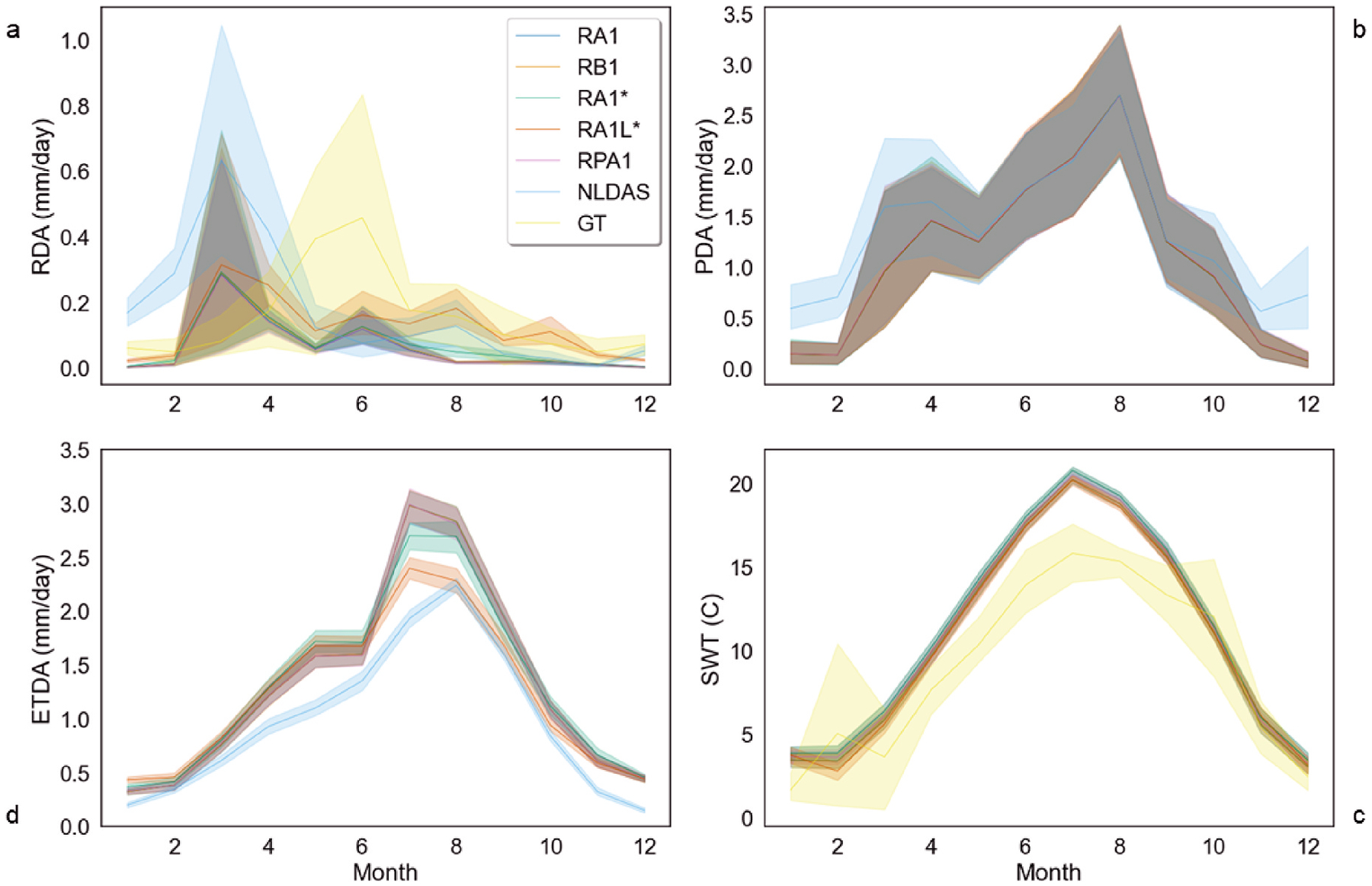
Modeled and observed monthly averaged water budget parameters and surface water temperatures line plots showing the 95% confidence interval (shaded). (*a*) Monthly averaged modeled runoff delineated average (RDA) comparison between VELMA, NLDAS, and observed values (GT); (b) Monthly averaged modeled precipitation delineated average (PDA) comparison between VELMA and NLDAS; (c) Monthly averaged modeled surface water temperatures (SWT) in degrees Celsius (°C) comparison between VELMA and GT; (d) Monthly averaged modeled actual evapotranspiration delineated average (ETDA) in comparison to NLDAS.

Similar seasonal patterns of the study simulated and NLDAS surface runoff exhibited rising limbs from February to March, falling limbs June–September, and base flow October to January, as observed by Rhoades et al. (2019) and Chow et al. (2019) (Fig. 3, Fig. 4a).

This seasonal pattern is reinforced in the 10-day and 30-day resampled mean runoff that closely resembled monitored data and NLDAS for simulations RA1L* & RA1* (Figs. 3 and 4a). Simulated evapotranspiration (ET) matched NLDAS (Fig. 4d) and daily estimates of actual evapotranspiration (AET) followed seasonal patterns observed in precipitation and surface runoff with similar rising and falling limbs (Fig. 4a and b). VELMA AET monthly averages performed better during the fall and winter in RA1L* in comparison with NLDAS. Monthly average precipitation between NLDAS and VELMA were comparable where major differences exist during the spring and winter (Fig. 4b). Annual declines in AET are observed between 2000 and 2006 in simulated values and NLDAS (See Appendix C.1).

### 4.2. Overall water quality and quantity

VELMA water quality parameters exhibited seasonal trends similar to observations with greater declines in RA1* and RPA1 compared to other simulations. Similar seasonal trends occurred in observed and modeled surface water temperatures (Fig. 4c). Simulated temperature averages were greater than observations but fell within the confidence intervals for all simulations. Observed surface water temperatures averaged 9.46 ± 5.69 °C, while modeled surface water temperature was 12.9% greater, averaging 10.7 ± 6.74 °C. Monthly observed average pH best matched simulation RA1L*. Simulated RA1L* pH was 4.02% greater than observed average pH of 7.93 during the disturbance interval. Greatest average of DOC and DON loss occurred in simulations RA1* and RPA1, corresponding to the VELMA large infiltration pre-fire and random priori post-fire wildfire initializations. RA1* and RPA1 DOC averages were almost two times greater than observed in RA1 and RB1. RA1L* DOC delineated concentration means were three orders of magnitude less than the other simulations, so are not shown in water quality comparisons.

**Fig. 5.**
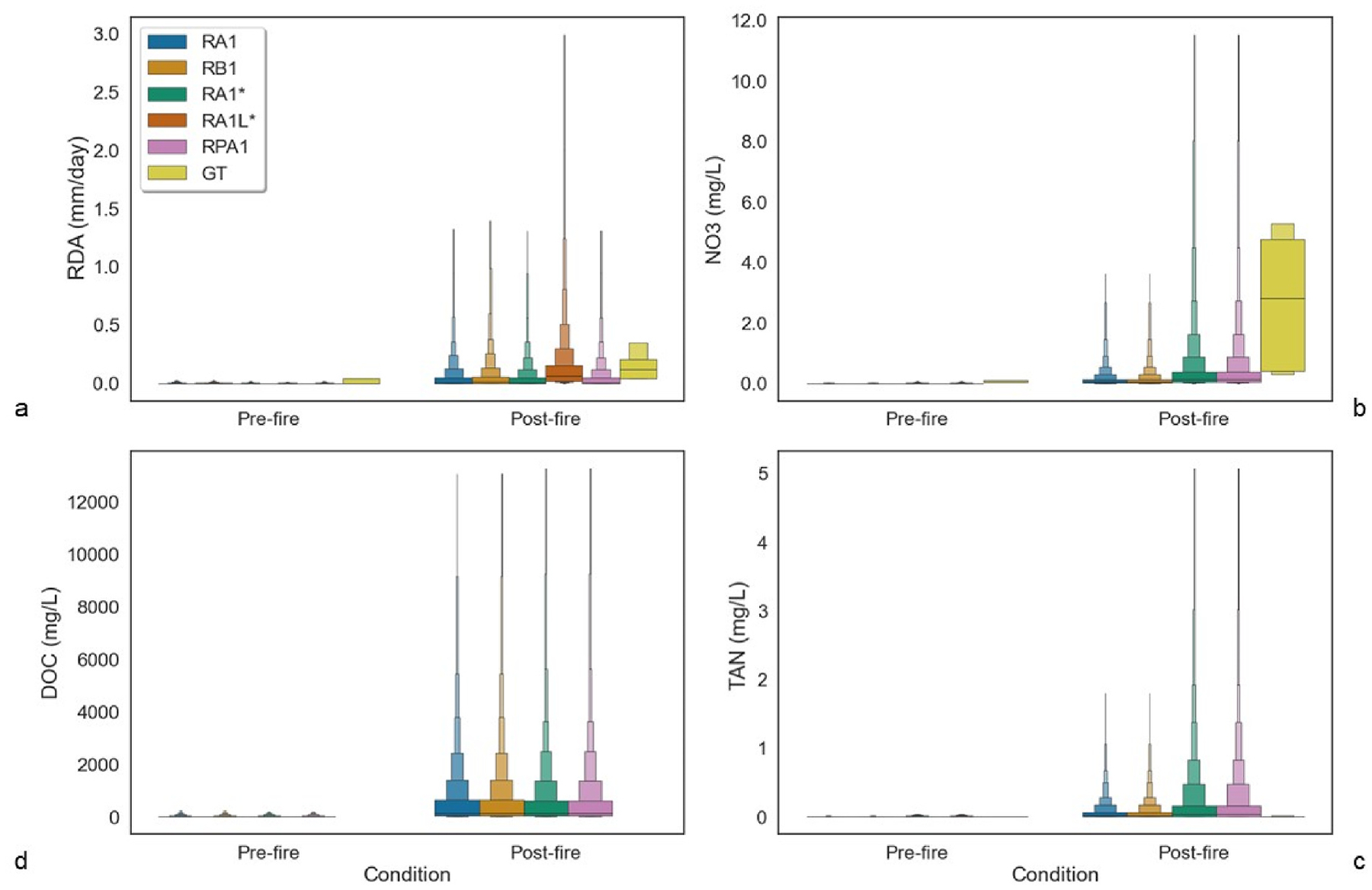
Pre-fire and post-fire comparison of modeled water quality parameters. (a) Boxen plot of simulated daily runoff average compared to observed from Brush Creek; (b) Boxen plot of simulated nitrate compared to observed (Measured as N); (c) Boxen plot of simulated TAN compared to observed (Measured as N) (d) Boxen plot of simulated DOC (No observed available) (Measured as C). Outliers for each boxen plot have been suppressed for the ease in interpolation.

Typically, VELMA underestimated nitrate concentrations during baseflow conditions. Greatest observed nitrate concentration averaged 2.44 mg N/L, less than double the concentrations in RA1* and RPA1 (Fig. 5b). The greatest nitrate concentration was observed at 9.72 mg N/L in comparison to the greatest modeled was over four times as much. Highest nitrate concentration simulated in RA1 and RB1 were only 0.459% different from the observed. TAN exhibited seasonal dynamics where greatest monthly average peaked during the months of April and June and declined during the fall and winter. Greatest TAN concentrations (1.23 × 10^1^ ± 0.596 mg N/L) were observed in simulation RA1* and RPA1 (Fig. 5c).

### 4.3. Pre-fire water quantity and quality

**Fig. 6.**
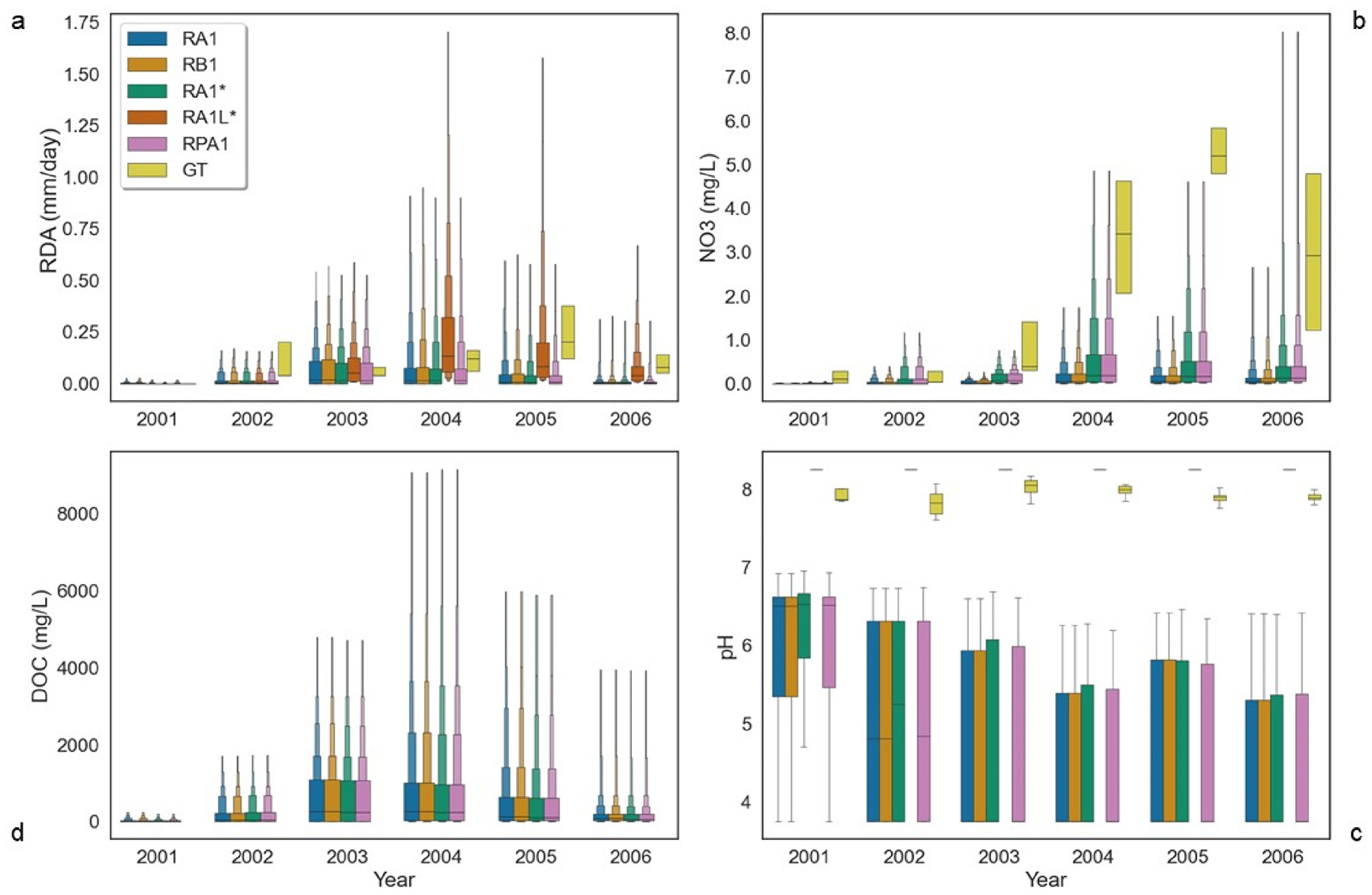
Annual disturbance interval comparison of modeled water quality parameters to observed. (a) Boxen plot of simulated daily runoff compared to observed; (b) Boxen plot of simulated nitrate compared to observed (Measured as N); (c) Box plot of simulated pH; (d) Boxen plot of simulated DOC (No observed available) (Measured as C). Outliers for each box plot have been suppressed for the ease in interpolation.

In general, we found limited differences and variability between simulations in pre-fire runoff and concentrations of nutrient daily losses (Figs. 5 and 6).

Simulated pre-fire daily surface runoff remained low during the disturbance interval. VELMA linked model (Table 2, Figs. 2e, and Fig. 5a) pre-fire runoff correlated well with observations. Greatest pre-fire daily averaged simulated runoff occurred in run RB1 followed by RA1* and RPA1. In both RA1 and RB1, average runoff (0.00236 mm per day) was an order of magnitude less than (0.0153 mm per day) observations. Simulated and observed pre-fire surface water temperature averaged 9.13 ± 6.62 °C. VELMA RA1L* modeled pH is most comparable to observed data by Rhoades et al. (2011), averaging between 7.8 for granitic basins to 8.2 for mixed geology basins (Fig. 6c). Modeled pH was also corroborated by an environmental impact statement for pre-fire conditions for a northern segment of the South Platte River from 1995 through 2002 (Colorado Department of Transportation & Federal Highway Administration, 2006) ranging between 7.4 and 8.5. Pre-fire observed average pH was 7.88, with simulated RA1L* average pH differing by 4.68% with limited variability (STD = 0.00) (Fig. 6c). The greatest pre-fire observed pH of 8.41 was only 1.9% less than the maximum VELMA pre-fire pH.

According to Rhodes et al. (2011), pre-fire water quality had elevated TN and was considered minimally disturbed (corresponding to 0.639% of VELMA and 25% of observed data) (Table 4 (US EPA, 2000a)). Pre-fire, simulated DOC average concentrations were similar between simulations RA1/RB1 and RA1*/RPA1, ranging from 7.81 × 10^−1^ to 4.89 × 10^1^ mg C/L (Fig. 5d). Pre-fire nitrate concentration were similar for simulations RA1/RB1 and RA1*/RPA1, averaging 2.20 × 10^−3^ mg N/L and 5.06 × 10^−3^ mg N/L in RA1*/RPA1 (Fig. 5b). All the observed nitrate values were classified as minimally disturbed. Collectively, between observed and VELMA, 10.3% of pre-fire nitrate levels of were minimally disturbed (Table 3, Fig. 6b). VELMA simulated and observed pre-fire nitrate concentrations were less variable than after the disturbance (Fig. 5b).

### 4.4. Post-fire water quantity and quality

Both VELMA simulated and observed data exhibited increased runoff and other water quantity shifts post-disturbance. Post-fire streamflow averaged 5270% more than pre-fire, with 0.109 mm of daily surface runoff. Best VELMA performance for surface runoff (average) in simulation RA1L* (0.169 mm per day) was 9.07% more than observed (0.169 mm per day) (Fig. 5a). VELMA simulations RA1* and RPA1 performed 47.6% less than observed daily surface runoff averages (Fig. 5a). Post-fire runoff averages for all simulations and observations increased monthly during the disturbance interval (Figs. 2e, 3, and 5a). VELMA post-fire ET observations were comparable to NLDAS (Fig. 4d) and correlated best with RA1L*. The daily ratio of AET to PET declined for all simulations post-fire and ranged between 0.110 and 0.534 because of AET loss due to vegetation decline. VELMA mean annual AET and rainfall plus snowmelt fractions performed best post-fire. A 29% decrease in annual precipitation occurred between 2000 and 2002 (Fig. 2b) between simulated values in VELMA and NLDAS.

Following fire, water quality declined in both the simulation and observed values relative to pre-fire conditions (Fig. 5). Post-fire surface water temperatures were 20.6% greater than pre-fire (See Appendix C.3). Post-fire observed surface water temperature averaged 10.1 ± 5.50 °C, differing between 8.45% and 10.8% in contrast to simulated SWT with an average of 11.0 ± 6.73 °C. Monthly post-fire SWTs means and 95th percentile ranges were greater than all pre-fire monthly averages except during November (no July pre-fire observed data were available), peaking June through September each year (Fig. 4c, See Appendix C.2). Simulated surface water temperature post-fire was comparable to Rhoades et al. (2011) seasonal averages ranging −7 to 24 °C. Applying Rhoades et al.’s three season four month averaging method, including winter, spring, and summer, all post-fire temperatures simulated and observed were higher than pre-fire conditions. The largest seasonal increases in post-fire SWT occurred during the winter (18.3%) and spring (20.7%) across all simulated and observed data. Post-fire pH was highly variable ranging from 3.75 to 8.25 (Fig. 6c). Average observed post-fire pH (7.94) differed by 3.91% (RA1L*).

Post-fire DOC average concentration was over 20 times greater than pre-fire (mean 4.63 × 10^2^ ± 1.44 × 10^3^ mg C/L) (Fig. 5d). VELMA RA1*/RPA1 post-fire average DOC was greatest of all simulations (6.85 × 10^2^ mg C/L), with a maximum concentration of 4.23 × 10^4^ mg C/L (Fig. 6d). Post-fire nitrate average concentration of 3.48 × 10^−1^ mg N/L was more than 79 times greater than pre-fire nitrate conditions across all simulations and observations. Observed average nitrate concentration increased 2480% compared to pre-fire (Fig. 5b). The greatest observed average nitrate concentration post-fire was 2.84 ± 2.43 mg N/L. In contrast, simulated average nitrate concentration was 82.6% less than observed with RA1*/RPA1 (4.94 ± 1.51 × 10^−1^ mg N/L). Maximum observed nitrate concentrations (9.72 mg N/L) were slightly larger (0.458%) in comparison with RA1/RB1. Annual averaged nitrate concentration increased at different rates depending on year post-fire (Fig. 6b). From 2004 through 2006, observed annual nitrate concentration was at least 78% greater than simulated; however, simulated results were within the 95% confidence interval (Fig. 6b) excluding 2005. Post-fire average TAN concentration (1.52 ± 0.487 × 10^−1^ mg N/L) was 3500% more than pre-fire (Fig. 5c). Post-fire average TAN simulated and observed values ranged between 8.02 × 10^−2^ (RA1/RB1) to 2.25 × 10^−1^ (RA1*/RPA1) mg N/L. Observed TAN concentration was almost 4 times less than simulated (RA1/RB1). Annual average TAN increased more rapidly in RA1* and RPA1 than other simulations.

### 4.5. Water quality criteria exceedance

**Fig. 7.**
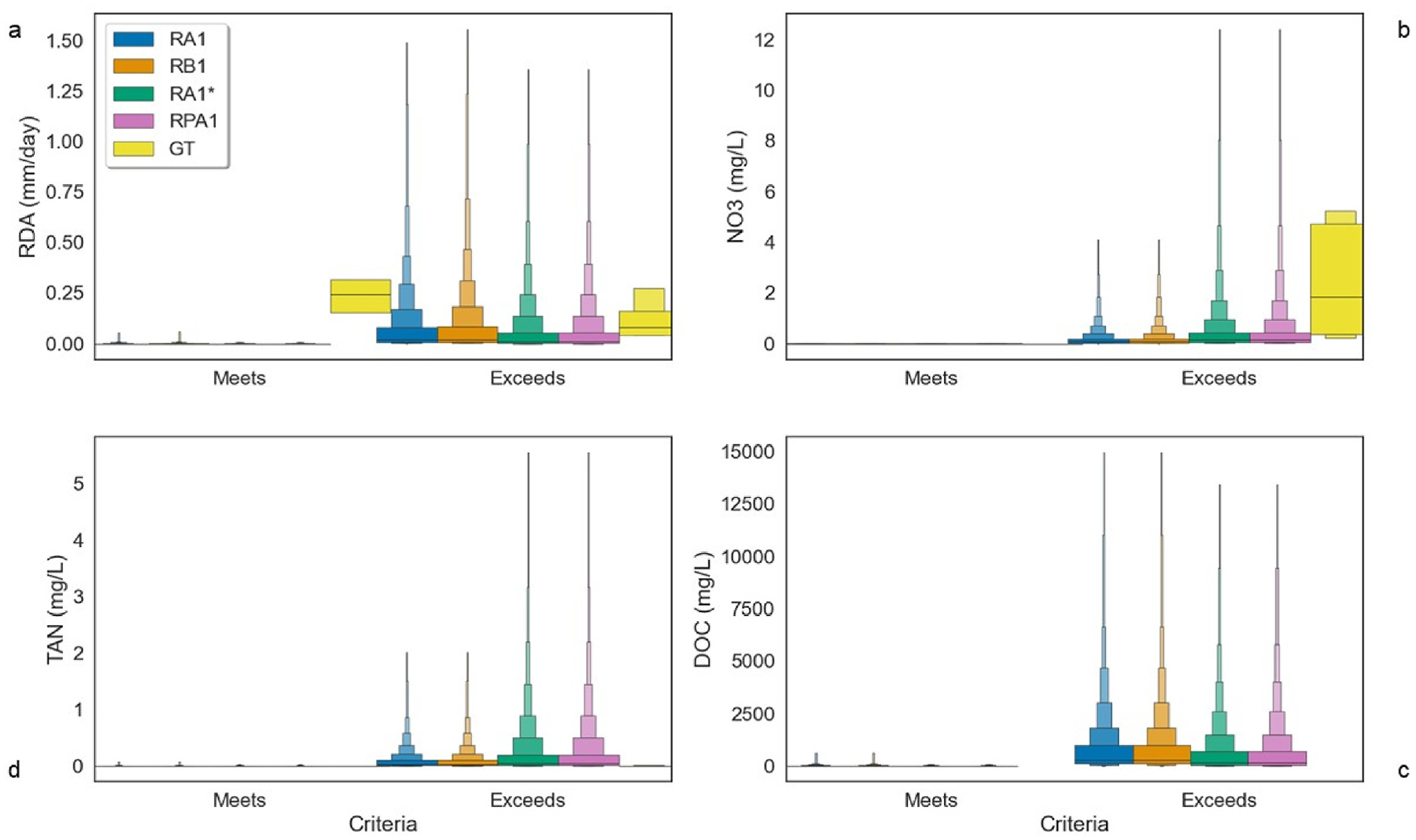
Comparison of modeled water quality exceedance criteria (shown without outliers) (Table 4); (a) Boxen plot of simulated daily runoff compared to observed (b) Boxen plot of simulated nitrate compared to observed (Measured as N); (c) Boxen plot of simulated DOC (No observed available) (Measured as C) (d) Boxen plot of simulated TAN compared to observed (Measured as N).

Less than 1% of simulated (RA1*/RPA1) or observed nitrate values exceeded the US EPA drinking water standard of 10 mg N/L (Table 4). However, several water quality standards were exceeded, and almost all post-fire simulated and observed data also exceeded the minimally disturbed streams criteria. Post-fire average nitrate concentrations between all simulated and observed data were 33 times greater than the 1.00 × 10^−2^ mg N/L aquatic life threshold (Figs. 6b and 7b).

Between the simulated and observed data analyzed, over 69% of the daily values met an exceedance criterion established (Table 4). For the least-disturbed streams of the Western Forested Mountains ecoregion, 40.7% of simulated and observed total nitrogen values were in exceedance (and 81.8% of observed data, Table 4). Higher simulated DOC concentrations, most prevalent in the spring, are mostly observed during exceedance events (Fig. 7c). Post-fire, DOC simulated levels are greater than Hohner et al.’s (2016) downstream South Platte River 0.2 mg/L DOC concentrations requiring higher alum dosage required to achieve optimal DOC removal during treatment.

Due to higher amount of simulated DOC, more acidic conditions are prevalent resulting in none of the TAN values exceeding the criteria according to the US EPA 1999 Ambient Water Quality Criteria (AWQC) for salmonids (US EPA 2013, Table 4) (Fig. 7d). Stream acidity simulated threatens to kill fish and smaller aquatic organisms. More than 3% of daily observations also exceeded Stressor Identification Guidance (SIG) (US EPA 2000b, Table 4). Most criteria exceedance occurrences were accompanied with higher nitrate, TAN, DOC, and runoff averaging 1.10 × 10^−1^ ± 9.92 × 10^−1^ mm per day (Fig. 7a).

## 5. Discussion and limitations

### 5.1. Discussion

The linked LANDIS-II and VELMA models successfully simulated the effects of wildfire on streamflow and water quality. Use of LANDIS-II to estimate forest community biomass and vegetation composition increased NSE post-fire, with best model performance after the disturbance. Spatial weather model calibrations using NLDAS climate drivers from two randomly selected locations with the delineated outlet performed well rather than use of the single outlet. Calibration performance improved by deploying larger infiltration rates and the LANDIS-II pre- and post-fire forest communities. The linked model’s topography, biomass, land coverage, and climate drivers supported calibration of the disturbance (Dlamini, 2010). Improvement of model performance was also attributed to the reduction of catchment size scale (Golden et al., 2014). Overall, the linked-modeling platform performed best during low flow events. At worst, model calibration performed as well as the mean of NLDAS data. Modeled differences occured during spring (March–June), where simulated surface runoff is increased due to earlier snowmelt than observed. Comparison of average monthly simulated runoff demonstrated elevated runoff post-fire, nominally an order of magnitude greater than pre-fire values in both observed and modeled results. This is likely associated with the high severity of the Hayman Fire (Rhoades et al., 2011; Fig. 2, Table 2) attributed to pre-fire homogenous forest densification (Fornwalt et al., 2016). Due to severity, post-fire recovery biomass regeneration continues to be limited (Chambers et al., 2016). Evidence of successful model calibration was also observed in precipitation, AET, PET, and surface water temperature.

Our simulations of increased runoff and declines in post-fire water quality are consistent with published literature and field campaigns. Reductions in forest biomass often lead to reduced infiltration and evapotranspiration rates, increasing overland flow and reducing groundwater recharge and baseflow (Paul et al., 2022; Shakesby and Doerr, 2006; Moody and Nyman, 2013; Morales et al., 2013; Murphy et al., 2018; Parlak, 2015). Surface water temperatures following disturbance are often greater due to the loss of the vegetative canopy, especially in the riparian zone (Minshall et al., 1989; Rhoades et al., 2011; Paul et al.). Both simulated and observed average water temperature increased 1.89 °C which can have drastic effects on fish habitats, especially for cold-water taxa (Rahel et al., 1996; Paul et al.). Loss of forest biomass also leads to reduced nitrogen demand, increased nutrient leaching, and greater in-stream nitrogen concentrations (Robinson and Minshall, 1991; Rhoades et al., 2011). Increases in post-fire DOC/DON and Particulate Organic Carbon have been observed and simulated (Bladon et al., 2008; Smith et al., 2011; Paul et al., 2022; Emelko et al., 2011; Murphy et al.; Writer et al., 2014). Nitrogen level increases may also be attributed to scorched foliage and sediment transported in moderate rain and melting events (Honher et al., 2016).

We observed distinct differences between the water quality in the RA1L* and other VELMA simulations. Simulation RA1L* exhibited greater correlations with NLDAS and observed surface runoff with a six-order less changes in forest biomass. Our other simulations deploy opposing traditional harvesting methods according to Rhoades et al. (2019), that remove most of the organic material generally resulting in less DOC and less impact on C/N ratios in streams and soils. Catchments with less biomass, as observed in simulation RA1L*, were affected less resulting in slightly basic water conditions.

Water quality degradation post-fire is consequential not only for aquatic habitats but also drinking water treatment, potentially leading to increased filtration, coagulant dosage, and biofouling. This can pose increased operational costs (Hohner et al., 2016) for providing drinking water in the near term, with potential long-term costs associated with reservoir dredging and maintenance of sedimentation tanks and clarifiers. Additionally, as surface water temperatures increase, the mixing of coagulants and disinfection agents may be altered due to changes in thermal equilibriums. Given the increased frequency and magnitude of wildfires and associated post-fire water quality degradation, infrastructure design and remediation budgets could be enhanced to better protect drinking water supplies and required treatment, especially during forest regeneration. This is inclusive of heterogeneity of stand, selective thinning, and more prescribed fires (Bartowitz et al., 2022; Hessburg et al., 2021). Reductions in forest biomass may also lead to uncontrolled or unpredictable streamflow, and forest regeneration and riparian management actions could also be taken to mitigate flooding in vulnerable subwatersheds.

### 5.2. Study limitations

There were several study limitations that may have affected our results. Due to limited availability of continuous, long-term climate and streamflow data, detailed calibration was limited to Brush Creek, rather than the entire AOI. Likewise, VELMA’s reliance on the HJA experimental forest for parametrization of biogeochemical pools and biomass fractions potentially limits its generalizability to other forests. Additionally, we may be underestimating water quality exceedances. Observations by Rhoades et al. (2011) were not continuous and did not include storm-related peak concentrations that may have exceeded the drinking water quality standard, given the likelihood of increased streamflow and nitrate concentrations during intense rainfall events (Larsen and MacDonald, 2007; Rhoades and Chow, 2017). Thresholds for pH calculations were increased to 8.5 rather than 8.25 (Park and Clough, 2018) to account for exceedance thresholds (Table 4), which may reduce reliability of measurements greater than 8.25. Finally, and more generally, although we have used the best available site characterization data to reconstruct the Hayman Fire, unknown site and event characteristics (e.g., differences in dynamic fire spread and fuel coefficients) limit our ability to simulate fire progression and associated water quality impacts. This is especially the case for the fire disturbance interval.

Because the linked-modeling platform is spatially explicit, it is feasible to evaluate the effectiveness of management actions and where these might be implemented best in the watershed. This includes both stand and riparian zone management as well as fuel management. We acknowledge these models are data and time intensive and hope to streamline the modeling process so that other researchers and managers could simulate the effects of management and wildfire in other watersheds with drinking water sources or particularly high-value or high-risk watersheds.

## 6. Conclusion

Wildfire activity in western US forests has increased over the last two decades, with accompanying risks to human life, property, air, and water quality. Water quality impacts are often less recognized, but wildfires mobilize solids and nutrient loadings to streams, and in some cases threaten drinking water supplies. Resource managers require spatially explicit, landscape modeling platforms to assess the potential impacts of fire on water quality and the efficacy of management practices such as thinning or prescribed burning. Modeling platforms can further provide the potential to link to multi-media tradeoffs, particularly air quality. This study has demonstrated the calibrated performance of the linked LANDIS-II -VELMA platform. Overall, our findings suggest researchers and managers can use this linked forest model to understand forest disturbances and potential management interventions to protect drinking water supplies. Our model linkage results agree with observations and literature relative to post-fire increases in surface runoff (3835%), surface water temperatures (20.6%), DOC (3387%), and nitrate concentrations 79 times greater than pre-fire conditions. Although few drinking water exceedances occurred, aquatic life stress was identified. This linkage provides a platform for integration with other grid-based models, such as those simulating air quality effects. Planned future research includes dynamic fire modeling via LANDIS-II and economic benefit/cost analysis for watershed management and post-fire drinking water treatment. Subsequently simplifying model input data pre-processing such that other researchers and managers can more easily implement for other watersheds is an additional consideration for wider adoption.

## Supplementary Material

Supplement1

Supplement2

Supplement3

## Figures and Tables

**Table 1:** LANDIS-II and VELMA input data sources. For details, please consult appendices A and B. (Abdelnour et al., 2013, Remillard, 1999, Smithwick et al., 2002, United States Environmental Protection Agency, 2021)

REQUIRED DATA	DATA SOURCES
**LANDIS-II Inputs**	
Ecoregions raster	US Department of Agriculture, Geospatial Data Gateway https://datagateway.nrcs.usda.gov/ Digital Elevation Model (DEM) – 30m Precipitation (1981–2010 PRISM derived annual average) ~ 800m Temperature (1981–2010 PRISM derived maximum and minimum averages) ~ 800m
	Multi-Resolution Land Characteristics (MRLC) Consortium https://www.mrlc.gov/ National Land Cover Database 2001 (NLCD 2001) – 30m
Initial forest communities raster	US Department of Agriculture Forest Service, Forest Inventory and Analysis (FIA) DataMart https://apps.fs.usda.gov/fia/datamart.html 2002–2005 forest tree inventory data 1984, 2002–2018 forest tree inventory data (See Appendix A for details.)
	US Geoloeical Survev. LANDFIRE https://www.landfire.gov/viewer/ Existing vegetation type layer v. 1.0.5 (2001) – 30m
	Multi-Resolution Land Characteristics (MRLC) Consortium https://www.mrlc.gov/ National Land Cover Database 2001 (NLCD 2001) – 30m
	US Department of Agriculture Forest Service, Design and Analysis Toolkit for Inventorv and Monitoring (DATIM) https://apps.fs.usda.gov/DATIM/Default.aspx Presence of species by ecoregion in the 2002–2005 analysis dataset.
	US Department of Agriculture Forest Service, ArcGIS Services https://apps.fs.usda.gov/fsgisx01/rest/services/RDW_FHP_TreeSpeciesMetrics Circa 2002 basal area rasters for individual species – 30m
	US Department of Agriculture, Geospatial Data Gateway https://datagateway.nrcs.usda.gov/ Digital Elevation Model (DEM) – 30m
	US EPA & US Geological Survey https://www.epa.gov/waterdata/nhdplus-national-hydrography-dataset-plus National Hydrography Dataset Plus (NHDPlus Version 2)
	Previously described ecoregions
Climate files	US Geological Survev, Geo Data Portal https://cida.usgs.gov/gdp/ 1980–2001 Daymet 1-km daily surface weather data (spin-up years) 2000–2002 Daymet 1-km daily surface weather data (fire simulation)
Species life history parameters; Biomass Succession Extension numeric parameters	A combination of online sources, personal communication, and the literature. Refer to Tables A.1 – A.7 for details.
Fire ecoregions raster	Ecoregions raster described above – 30m
	US Department of Agriculture Forest Service https://data.fs.usda.gov/geodata/edw/datasets.php National USFS Final Fire Perimeter geodatabase, 12/2022 refresh
	Geospatial Multi-Agency Coordination Group (GeoMAC) https://data-nifc.opendata.arcsis.com/ Historic Perimeters 2002
Dynamic Fire System and Dynamic Fuel System extensions numeric parameters	A combination of online sources, personal communication, and the literature. Refer to Tables B.1 – B.8 for details.
**VELMA Input Parameters**	
Area of Interest (AOI)	Retrieved using Google Earth Engine (GEE) https://code.earthengine.google.com/eb665ea903244ea50522cafe80058cca 30 meter Polygon based on main area of fire spread, watershed delineation, and model optimization using USGS NLCD 2001 and 2004 land cover difference
Elevation	National Aeronautics and Space Administration (NASA) Jet Propulsion Laboratory Shuttle Radar Topography Mission (SRTM) Version 3 Digital Elevation Model (DEM) (Farr et al., 2007). Obtained from Google Earth Engine https://code.earthengine.google.com/eb665ea903244ea50522cafe80058cca 30 meter 1 arc-second corrected for void applying a 1-gamma stretch
Watershed Delineation	Java Processing Digital Elevation Model (JPDEM), a DEM flat processing tool for watershed delineation (Pan et al., 2012) centrally focused on the Hayman Fire boundary NASA SRTM DEM Validated by National Hydrography Dataset Plus Version 2 (NHDPlus V2, US EPA & USGS, 2012) COMID (Common identifier of an NHD flowlines)
Vegetation	LANDIS-II Biomass Age and Species Cohorts (See Table 2 for more information on *, L) Pre-fire (2000*) Post-fire (2002) total aboveground biomass. Post-fire 6 orders of magnitude lower (L) biomass model representative of a high severity wildfire burning its fuel load
	Multi-Resolution Land Characteristics (MRLC) Consortium https://www.mrlc.gov/ National Land Cover Database 2001 (NLCD 2001) – 30 meter masking for vegetation or no vegetation classification
	H. J. Andrews (HJA) Experimental Ponderosa Pine Forest 400-year biomass succession old-growth values. Smithwick et al. (2002) Remillard (1999) Abdelnour et al. (2013)
	Carbon and nitrogen balances controlling succession and net primary production partitioned between the Leaf, Root, and Stem (LRS) and detritus spatially and temporally above and below ground. McKane et al., 2014
Soil Map	US General Soil Map (STATSG02) (USDA Natural Resources, 2016; Meinke, 2004; McKane et al., 2022) for the state of Colorado Clipped and masked by waterbodies for the AOI
Land Use Scenario	US Geological Survey (USGS) & US Forestry Service (USFS) 2014 Hayman Fire boundary polygon as a mask. Applied LRS disturbance filter based on Monitoring Trends in Bum Severity (MTBS) shapefile for fire disturbance based on burned vs unbumed classification areas in the AOI
Climate	North American Land Data Assimilation (NLDAS) daily averaged temperature (K) and precipitation (mm) from the US Environmental Protection Agency (US EPA) (2020 – 2021) Hydrologic Micro Services (HMS) 1992–2019 Kelvin (K) converted to Celsius (° C) VELMA spatial weather model was initialized by 3 locations which were randomly (R) selected and randomly selected using priori knowledge (RP) (See Table 2 for more information on R, RP) **Table:** Index Name Coordinates Cell Indices Models Used Outlet Index_293321 (39.263°, −105.227°) (394, 579) All Index_261345 (39.275°, −105.236°) (351, 552) Random (R) Index_250888 (39.278°, −105.255°) (337, 497) R Index_205535 (39.295°, −105.266°) (276, 467) Random Priori (RP) Index_275461 (39.269°, −105.237°) (370, 551) RP
Validation Hydrology	US EPA HMS NLDAS daily surface runoff for each corresponding NHDPlus V2 COMID 1992 – 2019 COMID (190641); (39.263°, −105.227°)

**Table 2:** Brush Creek simulation branches denoted by the following attributes: (1 = Fire; RP = Random Priori Weather Driver Selection; R = Random Weather Driver Selection; L- Less AG; * = LANDIS Pre-fire (2000) conditions). This table shows the best runs calibrated the with Nash-Sutcliffe Efficiency (NSE) coefficient and Root Mean Square Error (RMSE) utilizing a 95% biomass mortality through a leaf-root-stem (LRS) harvest incorporating a 10% offsite removal for the wildfire disturbances simulated. Harvested biomass offsite percentage and soil infiltration rates (See Table 3 for more information; A – Larger Infiltration Rates; B – Lower Infiltration Rates) are also described. LANDIS Pre-fire simulation runs are denoted by the asterisk. The Parent run is performed using a single weather station to model the Brush Creek watershed delineation.

RUN	NSE	RMSE	Total AG Biomass (g of C)	Increased *K*_*s*_, *f*_v_, *f*_l_
Parent	0.413	0.754	2.95E+09	YES^A^
RA1	0.609	0.615	2.95E+09	YES^A^
RB1	0.609	0.616	2.95E+09	NO^B^
RPA1	0.601	0.622	2.95E+09	YES^A^
LANDIS-II (Pre-fire Branch*)				
RA1L* (High Severity Burn - L)	0.621	0.606	3.21E+03	YES^A^
RA1*	0.612	0.613	3.13E+09	YES^A^

**Table 3:** Model parameter values used to simulate the hydrologic processes and groundwater transport in the Hayman Fire. Three soil textures (loam, sandy loam, and water) were calibrated from the soil(s) database within the AOI. A and B parameters refer to model initialization calibration differences using faster and slower soil infiltration rates.

Model Parameters Values Used to Simulate the Hydrologic Processes in the Hayman Fire			
Parameters	Definition	Value	References
Soil texture	dominant soil texture	loam	Abdelnour et al. (2011); McKane et al. (2014)
*K*_*s*_	surface saturated hydraulic conductivity (mm day^−1^)	317	Abdelnour et al. (2011); McKane et al. (2014)
*K*_*sv*_	Vertical surface saturated hydraulic conductivity exponential decay factor	0.004	*calibrated*
*K*_*sl*_	Lateral surface saturated hydraulic conductivity exponential decay factor	0.00325^A^	*calibrated*
		0.00310^B^	
$\theta_{i}^{fc}$	field capacity in layer i	0.27	Abdelnour et al. (2011); McKane et al. (2014)
*φ*_*i*_	porosity in layer i	0.463	Abdelnour et al. (2011); McKane et al. (2014)
$\theta_{i}^{w}$	wilting point in layer i	0.117	Abdelnour et al. (2011); McKane et al. (2014)
*ρ*_*b*_	bulk density (grams cm^−3^)	1.42	Abdelnour et al. (2011); McKane et al. (2014)
Soil texture	dominant soil texture	sandy loam	Abdelnour et al. (2011); McKane et al. (2014)
*K*_*s*_	surface saturated hydraulic conductivity (mm day^−1^)	622	Abdelnour et al. (2011); McKane et al. (2014)
*K*_*sv*_	Vertical surface saturated hydraulic conductivity exponential decay factor	0.005	*calibrated*
*K*_*sl*_	Lateral surface saturated hydraulic conductivity exponential decay factor	0.00325^A^	*calibrated*
		0.00310^B^	
$\theta_{i}^{fc}$	field capacity in layer i	0.207	Abdelnour et al. (2011); McKane et al. (2014)
*φ*_*i*_	porosity in layer i	0.453	Abdelnour et al. (2011); McKane et al. (2014)
$\theta_{i}^{w}$	wilting point in layer i	0.095	Abdelnour et al. (2011); McKane et al. (2014)
*ρ*_*b*_	bulk density (grams cm^−3^)	1.52	Abdelnour et al. (2011); McKane et al. (2014)
Soil texture	dominant soil texture	water	*calibrated*
*K*_*s*_	surface saturated hydraulic conductivity (mm day^−1^)	6000	*calibrated*
*K*_*sv*_	Vertical surface saturated hydraulic conductivity exponential decay factor	0.005	*calibrated*
*K*_*sl*_	Lateral surface saturated hydraulic conductivity exponential decay factor	0.00325^A^	*calibrated*
		0.00310^B^	
$\theta_{i}^{fc}$	field capacity in layer i	0.207	Abdelnour et al. (2011); McKane et al. (2014)
*φ*_*i*_	porosity in layer i	0.9	*calibrated*
$\theta_{i}^{w}$	wilting point in layer i	0.095	Abdelnour et al. (2011); McKane et al. (2014)
*ρ*_*b*_	bulk density (grams cm^−3^)	1.05	*calibrated*
pH		7.5	*calibrated*
**Preharvest Calibrated Model Parameters**			
Δz_1–4_	soil surface layer (layers 1–4) thickness (mm)	1651	Adopted from Price and Amen (1984)
Δz_1_	soil surface layer (layer 1) thickness weight	0.15	*calibrated*
Δz_2_	first intermediate soil surface layer (layer 2) thickness weight	0.375	*calibrated*
Δz_3_	second intermediate soil surface layer (layer 3) thickness weight	0.375	*calibrated*
Δz_4_	deep soil surface layer (layer 4) thickness weight	0.1	*calibrated*
*K*_s_	surface saturated hydraulic conductivity (mm day^−1^)	18000	*calibrated*
*f*_v_	vertical exponential decay factor	0.009	*calibrated*
*f*_1_	lateral exponential decay factor	0.007	*calibrated*
pH		6.5	Retzer (1949)
	biomass mortality fraction through an AG and below ground leaf-root-stem (LRS) harvest	0.95	*calibrated*
	biomass offsite fraction removal from AG and below ground LRS	0.1	*calibrated*

* Soil texture varied between (Loam, Fine Sandy Loam, and Coarse Sandy Loam).

*A – Larger Infiltration Rates;

B – Lower Infiltration Rates.

**Table 4:** Water quality parameters criteria for exceedance and references. The water quality exceedance criteria included aquatic stress, drinking water compliance, stream disturbance, and ambient water quality criteria for simulated and observed parameters.

Water Quality Parameters Criteria (mg/L) for Exceedances			
Parameters	Criteria	Notation	References
Ammonia (Measured as Nitrogen)	0.57 at pH 8.5	*NH*_3_	US EPA (2000b)
	1.27 at pH 8.0		
Nitrate (Measured as Nitrogen)	10, 1.6, 0.014, 0.01	*NO*_3_	US EPA (2000a,b, 1986)
			Rankin et al. (1999)
			Chatfield Watershed Authority (2015)
			Rhoades et al. (2011, 2019)
Total Phosphorus^c^	0.28	*PO*_4_	US EPA (2000b)
			Rankin et al. (1999)
Total Ammonia as Nitrogen (Measured as Nitrogen)	5.6 at pH 8.0^a^	*TAN*	US EPA (2013)
	24 at pH ≥ 7.0 &≥20 °C^a^		
	2.9 at pH ≥ 8.0 &≥25 °C^b^		
	19 at pH ≥ 7.0 &≥20 °C^b^		
	17 at pH≥7.0 &≥20 °C^a^		
Total Nitrogen (Measured as Nitrogen)	0.12, 1.25	*TN*	US EPA (2000a)
			Rhoades et al. (2011, 2019)
			Colorado (2016)

a Salmonids Present.

b Mussels Present.

c Only examined for Ground Observations.

## Data Availability

Data will be made available on request.

## Abbreviations:

AG: aboveground
AET: Actual Evaporation
AWQC: Ambient Water Quality Criteria
AOI: area of interest
COMID: Common identifier of an NHD flowline
DATIM: Design and Analysis Toolkit for Inventory and Monitoring
DEM: Digital Elevation Model
DBPs: disinfection byproducts
DOC: Dissolved Organic Carbon
DON: Dissolved Organic Nitrogen
ETDA: Actual Evapotranspiration Delineated Average
GT: Ground Truth
HJA: H.J. Andrews
HMS: Hydrologic Micro Services
HUC: hydrologic unit code
LANDIS: LANDscape DIsturbance and Succession
LRS: leaf-root-stem
NSE: Nash-Sutcliffe Efficiency
NHDPlus V2: National Hydrography Dataset Plus Version 2
NAD: North American Datum
NLDAS: North American Land Data Assimilation System
PET: Potential Evapotranspiration
PDA: Precipitation Delineated Average
RMSE: Root Mean Square Error
RDA: Runoff Delineated Average
SIG: Stressor Identification Guidance
SWT: Surface Water Temperature
TAN: Total Ammonia as Nitrogen
TN: Total Nitrogen
USDA: United States Department of Agriculture
USFS: United States Forest Service
USGS: United States Geological Survey
UTM: Universal Transverse Mercator
US EPA: US Environmental Protection Agency
VELMA: Visualizing Ecosystem Land Management Assessments
WUI: wildland urban interface (WUI)
