# Supplementary material for: Model linkage to assess forest disturbance impacts on water quality: A wildfire case study using LANDIS(II)-VELMA
Source: Environ Model Softw. Author manuscript; Available in PMC 2025 Sep 1. (PMC11457591; doi:10.1016/j.envsoft.2024.106134)
Supplement: Supplement1 [file NIHMS2016776-supplement-Supplement1.pdf]

Journal: Environmental Modelling & Software

Title: Model linkage to assess forest disturbance impacts on water quality: a wildfire case study using LANDIS(II)-VELMA

Authors: Kar'retta Venable, John M. Johnston, Stephen D. LeDuc, and Lourdes Prieto

Appendix C: VELMA Additional Water Budget and Surface Water Temperature Analysis

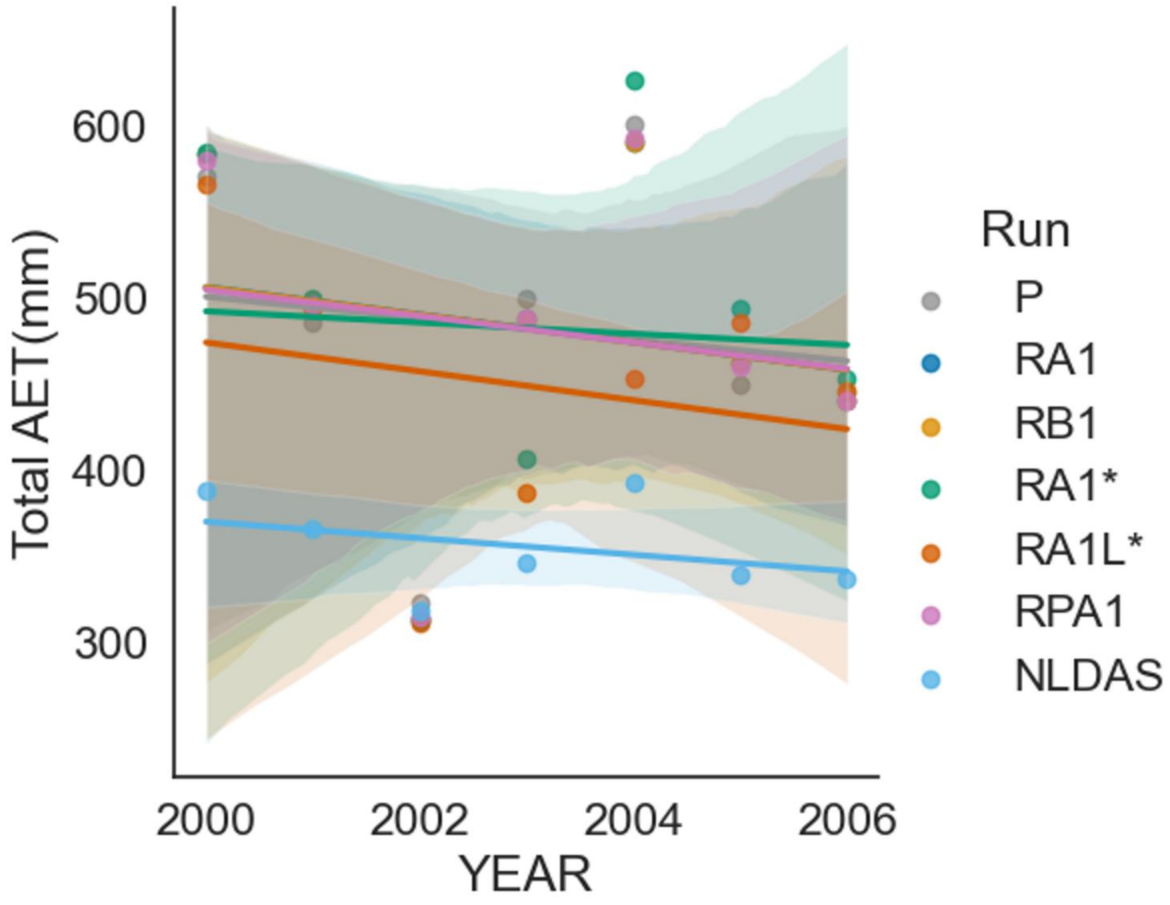

Figure. C.1 – Plotted data and regression model line plots with 95% confidence interval (shaded) annual Actual Evapotranspiration Totals (AET) in millimeters (mm) for each simulation run in comparison to NLDAS.

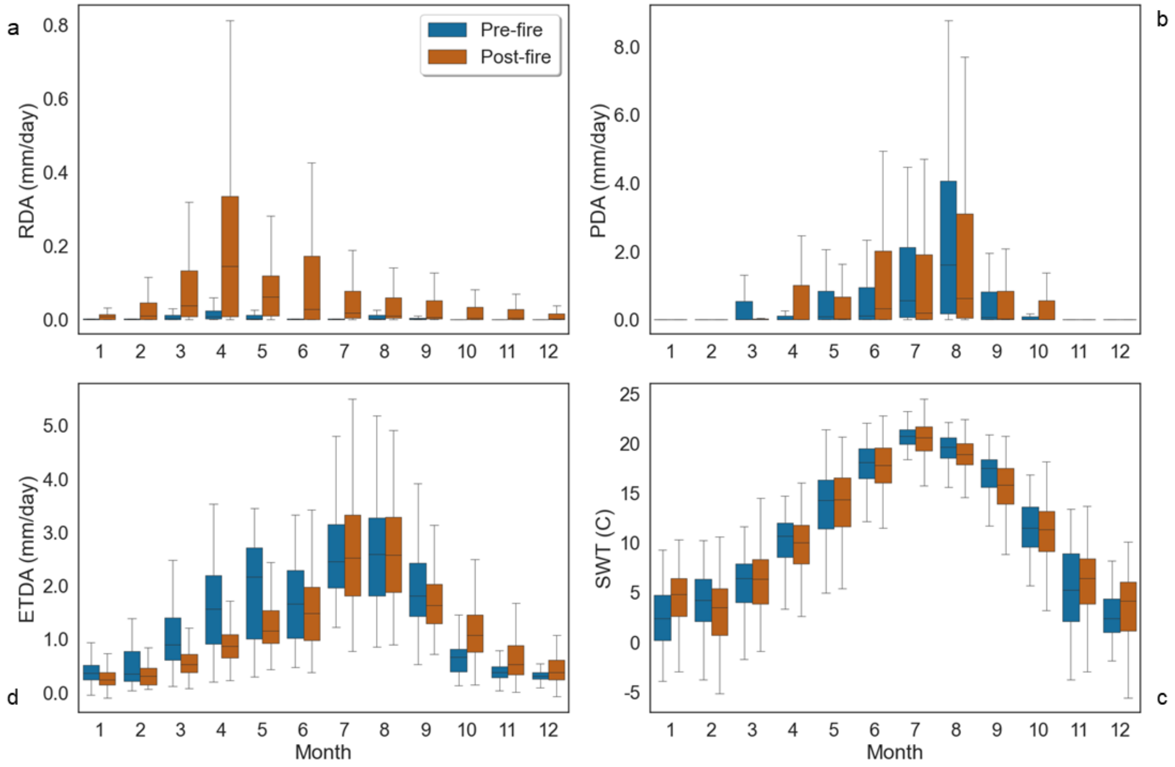

Fig. C.2 Pre-fire and post-fire monthly averaged comparison of modeled and observed averaged water budget parameters and surface water temperatures box plots. (*a*) Modeled runoff delineated average (RDA); (b) Modeled precipitation delineated average (PDA); (c) Modeled surface water temperatures (SWT) in degrees Celsius (°C); (d) Modeled actual evapotranspiration delineated average (ETDA).

Journal: Environmental Modelling & Software

Title: Model linkage to assess forest disturbance impacts on water quality: a wildfire case study using LANDIS(II)-VELMA

Authors: Kar'retta Venable, John M. Johnston, Stephen D. LeDuc, and Lourdes Prieto

Appendix C: VELMA Additional Water Budget and Surface Water Temperature Analysis

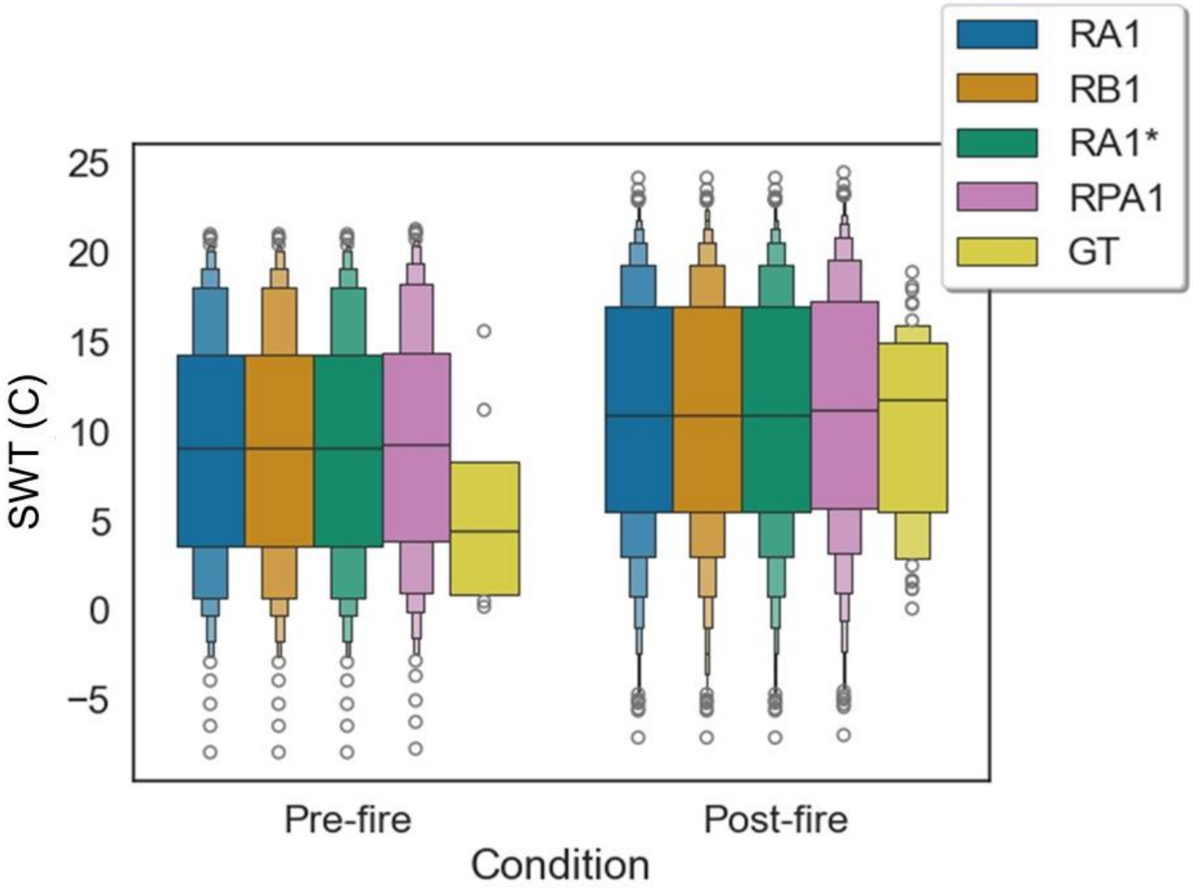

Fig. C.3 Pre-fire and post-fire comparison of modeled and observed surface water temperatures (SWT) in degrees Celsius (℃) boxen plots.
