# Supplementary material for: Model linkage to assess forest disturbance impacts on water quality: A wildfire case study using LANDIS(II)-VELMA
Source: Environ Model Softw. Author manuscript; Available in PMC 2025 Sep 1. (PMC11457591; doi:10.1016/j.envsoft.2024.106134)
Supplement: Supplement2 [file NIHMS2016776-supplement-Supplement2.pdf]

Journal: Environmental Modelling & Software

Title: Model linkage to assess forest disturbance impacts on water quality: a wildfire case study using LANDIS(II)-VELMA

Authors: Kar'retta Venable, John M. Johnston, Stephen D. LeDuc, and Lourdes Prieto

Appendix B: LANDIS-II fire extension input parameters

This appendix contains the input parameters values (Tables B.1 - B.8) and the fire ecoregions (Figure B.1.) used to simulate the 2002 Colorado Hayman Fire using LANDIS-II Dynamic Fire System and Dynamic Fuel System extensions (version 3.0) (Sturtevant et al., 2009). As the tables indicate, sources consulted to help determine parameter values included the literature, online material, and personal communication. For more information on these parameters, please consult the extensions user guides (http://www.landis-ii.org/extensions). Details on the parameters used by the succession extension during the simulation can be found in Appendix A.

The fire ecoregions (Fig. B1) were derived from the project's ecoregions (Fig. A.1). The montane dry and montane mesic ecoregions were combined into the moderate elevation ecoregion. The ecoregions were then clipped by the Hayman fire perimeter and the areas that should not burn were identified. This constrained the burning to the inside of the Hayman perimeter. The fire perimeter used was derived from a United States Department of Agriculture, Forest Service (2022) feature class. We shifted this perimeter about 213 meters due north to approximate the location of a Hayman fire outline from the Geospatial Multi-Agency Coordination Group (GeoMAC, n.d.), which better aligned with ancillary sources like satellite/aerial imagery.

During calibration, the extensions' parameters (Tables B.1 - B.8) were adjusted until the total biomass inside the Hayman fire perimeter was within 1% (0.12% difference) of the biomass estimates derived from the year 2002 band of the LandTrendr Biomass, CONUS (1990-2017) dataset (Hooper and Kennedy, 2018). See Fig. B.2 for a depiction of the aboveground biomass in the study areas after the simulation of the Hayman Fire.

Tables B.1 - B.8. Where applicable, superscript numbers indicate the sources consulted to determine parameter value.

Table B.1. Species fuel coefficients table for LANDIS-II Dynamic Fuel System Extension.

Species Code	Fuel Coefficient[1-3]
abielasi	1.0
juniscop	1.0
piceenge	1.0
picepung	1.0
pinuaris	1.0
pinucont	1.0
pinupond	1.0
pinuflex	1.0
pseumenz	1.0
poputrem	1.0
quergamb	1.0
shrubs	1.0

[1]Scheller & Cassell (2019a)
[2]Scheller & McCauley (2020b)
[3]Vukomanovic (2020)

Table B.2. Hardwood maximum and dead fir maximum age parameters for LANDIS-II Dynamic Fuel System Extension.

Hardwood Maximum (%)[1]	Dead Fir Maximum Age (years)[1]
20	25

[1]Scheller & Cassell (2019a)

Table B.3. Fuel type table for LANDIS-II Dynamic Fuel System Extension. Values reflect adjustments made during calibration. As a result, some values are outside the ranges seen in the sources consulted.

Fuel Type Index	Base Fuel Type	Age Range (years)[1-8]	Species Code
1	Conifer	0 to 50	abielasi
2	Conifer	51 to 100	abielasi
3	Conifer	101 to 1000	abielasi
4	Conifer	0 to 50	piceenge
5	Conifer	51 to 100	piceenge
6	Conifer	101 to 1000	piceenge
7	Conifer	0 to 40	picepung
8	Conifer	41 to 80	picepung
9	Conifer	81 to 1000	picepung
10	Conifer	0 to 60	pinuflex
11	Conifer	61 to 130	pinuflex
12	Conifer	131 to 1000	pinuflex
13	Conifer	0 to 60	pinuaris
14	Conifer	61 to 130	pinuaris
15	Conifer	131 to 1600	pinuaris
16	Conifer	0 to 60	pinucont
17	Conifer	61 to 130	pinucont
18	Conifer	131 to 1000	pinucont
19	Conifer	0 to 60	pseumenz
20	Conifer	61 to 80	pseumenz
21	Conifer	81 to 1000	pseumenz
22	Conifer	0 to 50	pinupond
23	Conifer	51 to 80	pinupond
24	Conifer	81 to 1000	pinupond
25	Conifer	0 to 60	pinupond pseumenz
26	Conifer	61 to 80	pinupond pseumenz
27	Conifer	81 to 1000	pinupond pseumenz
28	Conifer	0 to 50	juniscop
29	Conifer	51 to 100	juniscop
30	Conifer	101 to 1000	juniscop
31	Deciduous	0 to 40	poputrem
32	Deciduous	41 to 80	poputrem
33	Deciduous	81 to 200	poputrem
34	Deciduous	0 to 35	quergamb
35	Deciduous	36 to 150	quergamb
36	Conifer	0 to 80	shrubs
37	Open	0 to 10	shrubs

[1]Colorado State Forest Service (n.d.)
[2]Creutzburg et al. (2017)
[3]Moench (2006)
[4]Scheller (2016b)
[5]Scheller & Cassell (2019a)
[6]Scheller & Kretchun (2017b)
[7]Scheller & McCauley (2020b)
[8]Vukomanovic (2020)

Table B.4. Ecoregion-dependent parameters table for LANDIS-II Dynamic Fire System Extension. Values reflect adjustments made during calibration. As a result, some values are outside the ranges seen in the sources consulted. Sources consulted are listed at the bottom of the table.

Ecoregion Map Code	Ecoregion Name	Mu	Sigma	Maximum Duration (minutes)	Spring FMC Low (%)	Spring FMC High (%)	Spring High Proportion	Summer FMC Low (%)	Summer FMC High (%)	Summer High Proportion	Fall FMC Low (%)	Fall FMC High (%)	Fall High Proportion	Open Fuel Type Index	Number of Ignitions
1	Mod	8.88	6.58	43200	85	120	0.05	85	100	0.5	75	90	0.1	37	600
2	High	8.88	6.58	43200	100	130	0.05	85	100	0.5	75	90	0.1	37	400
3	Low	8.88	6.58	43200	85	120	0.05	85	100	0.5	75	90	0.1	37	700
4	Inactive	0	0	0	0	0	0	0	0	0	0	0	0	0	0
0	NoData	0	0	0	0	0	0	0	0	0	0	0	0	0	0

FMC = foliar moisture content

Creutzburg et al. (2017)
Scheller (2016a)
Scheller & Cassell (2019c)
Scheller & Kretchun (2017a)
Scheller & McCauley (2020a)
Vukomanovic (2020)

Table B.5. Seasons table for LANDIS-II Dynamic Fire System Extension.

Season Name	Leaf Status	Proportion of Fires*	Percent Grass Curing (%)[1-2]	Daylength Proportion
Spring	LeafOff	0	50	1.0
Summer	LeafOn	1	100	1.0
Fall	LeafOff	0	100	1.0

*Only summer fires were simulated.

[1]Scheller (2016a)
[2]Scheller & McCauley (2020a)

Table B.6. Fuel type table for LANDIS-II Dynamic Fire System Extension. Values reflect adjustments made during calibration. As a result, some values are outside the ranges seen in the sources consulted. Sources consulted are listed at the bottom of the table.

Fuel Type Index	Base Fuel Type	Surface Type	Ignition Probability	a	b	c	q	Buildup Index (BUI)	Maximum Buildup Effect	Crown Base Height (CBH) (meters)
1	Conifer	C2	1	100	0.0282	1.5	0.75	64	1.321	1
2	Conifer	C2	1	80	0.0282	3	0.85	74	1.321	2
3	Conifer	C2	1	80	0.0282	3	0.85	74	1.321	2
4	Conifer	C2	1	70	0.0282	1.5	0.5	72	1.321	1
5	Conifer	C2	1	50	0.0282	4.0	0.90	120	1.321	2
6	Conifer	C2	1	50	0.0282	4.5	0.99	120	1.321	3
7	Conifer	C5	1	35	0.0697	4	0.9	56	1.22	1
8	Conifer	C5	1	30	0.0697	4	0.8	56	1.22	1
9	Conifer	C5	1	30	0.0697	4	0.8	56	1.22	2
10	Conifer	C2	1	60	0.0282	1.5	0.7	64	1.321	1
11	Conifer	C7	1	40	0.0305	2.0	0.85	106	1.134	4
12	Conifer	C7	1	40	0.0305	2.0	0.85	106	1.134	4
13	Conifer	C2	1	45	0.0282	1.5	0.85	106	1.321	1
14	Conifer	C7	1	45	0.0305	4	0.8	106	1.134	3
15	Conifer	C7	1	45	0.0305	4	0.8	106	1.134	4
16	Conifer	C2	1	100	0.0282	1.5	0.7	62	1.321	1
17	Conifer	C6	1	100	0.08	3.5	0.7	62	1.197	2
18	Conifer	C6	1	100	0.08	3.5	0.7	62	1.197	5
19	Conifer	C2	1	30	0.0282	1.5	0.7	64	1.321	1
20	Conifer	C2	1	40	0.0282	1.5	0.7	64	1.321	2
21	Conifer	C3	1	40	0.0444	3.0	0.8	100	1.261	5
22	Conifer	C2	1	10	0.0282	4.5	0.9	85	1.321	1
23	Conifer	C3	1	15	0.0444	3.0	0.75	62	1.261	2
24	Conifer	C3	1	10	0.0444	4.5	0.99	90	1.261	5
25	Conifer	C7	1	10	0.0305	4.5	0.85	106	1.134	1
26	Conifer	C7	1	45	0.0305	3.0	0.85	106	1.134	2
27	Conifer	C7	1	45	0.0305	3.0	0.85	106	1.134	5

Table B.6. Fuel type table for LANDIS-II Dynamic Fire System Extension, Continued

Fuel Type Index	Base Fuel Type	Surface Type	Ignition Probability	a	b	c	q	Buildup Index (BUI)	Maximum Buildup Effect	Crown Base Height (CBH) (meters)
28	Conifer	C2	1	10	0.0282	4.0	0.95	90	1.321	1
29	Conifer	C2	1	10	0.0282	3.5	0.95	64	1.321	2
30	Conifer	C2	1	10	0.0282	3.5	0.95	64	1.321	4
31	Deciduous	D1	1	70	0.0232	1.6	0.70	30	1.179	1
32	Deciduous	D1	1	100	0.0232	1.6	0.60	30	1.179	2
33	Deciduous	D1	1	25	0.0232	1.6	0.60	32	1.179	4
34	Deciduous	D1	1	30	0.0232	1.6	0.9	40	1.179	1
35	Deciduous	D1	1	30	0.0232	1.6	0.9	40	1.179	2
36	Conifer	C2	1	5	0.0282	4.0	0.7	64	1.321	1
37*	Open	O1a	0.5	8	0.02	4.5	0.7	120	1.076	0
38	Conifer	M1	1	0	0	0	0.8	50	1.250	3
39	Conifer	M2	1	0	0	0	0.8	50	1.250	3

*We commented out this fuel type to keep those cells from burning.

Creutzburg et al. (2017)
Scheller (2016a)
Scheller & Cassell (2019b)
Scheller & Kretchun (2017a)
Scheller & McCauley (2020a)
Sturtevant et al. (2009)
Vukomanovic (2020)

Table B.7. Fire damage table for LANDIS-II Dynamic Fire System Extension.

Cohort Ages Killed (% of species longevity)[1]	Fire Severity – Species Fire Tolerance Differential[1]
5%	-2
10%	-1
30%	0
50%	1
80%	2
100%	3

For Fire severity 1 represents the lowest severity and 5 the highest.
For Fire tolerance 1 represents the lowest tolerance and 5 the highest.

[1]Jung et al. (2023)

Table B.8. Initial weather table for LANDIS-II Dynamic Fire System Extension. Values reflect adjustments made during calibration. Sources are listed at the bottom of the table.

Fine Fuel Moisture Code (FFMC)	Buildup Index (BUI)	Wind Speed Velocity (km/hr)	Wind Direction* (degrees)	Fire Weather Index class**	Season	Ecoregion Name
95.59194628	130.8184446	22.65956352	25	1	summer	High
95.59194628	130.8184446	22.65956352	25	2	summer	High
95.59194628	130.8184446	22.65956352	25	3	summer	High
95.59194628	130.8184446	22.65956352	25	4	summer	High
95.59194628	130.8184446	22.65956352	25	5	summer	High
95.59194628	130.8184446	22.65956352	25	1	summer	Mod
95.59194628	130.8184446	22.65956352	25	2	summer	Mod
95.59194628	130.8184446	22.65956352	25	3	summer	Mod
95.59194628	130.8184446	22.65956352	25	4	summer	Mod
95.59194628	130.8184446	22.65956352	25	5	summer	Mod
95.234368	130.8184446	22.65956352	25	1	summer	Low
95.234368	130.8184446	22.65956352	25	2	summer	Low
95.234368	130.8184446	22.65956352	25	3	summer	Low
95.234368	130.8184446	22.65956352	25	4	summer	Low
95.234368	130.8184446	22.65956352	25	5	summer	Low

*Direction wind is blowing TO.
**No distinction between classes was made while simulating the Hayman Fire.

Loudermilk (2020) – Python script *FireWeatherIndex_Calculations.py*
Western Regional Climate Center (2020) – 2002 weather data from the Cheeseman, Colorado Remote Automated Weather Station (RAWS)

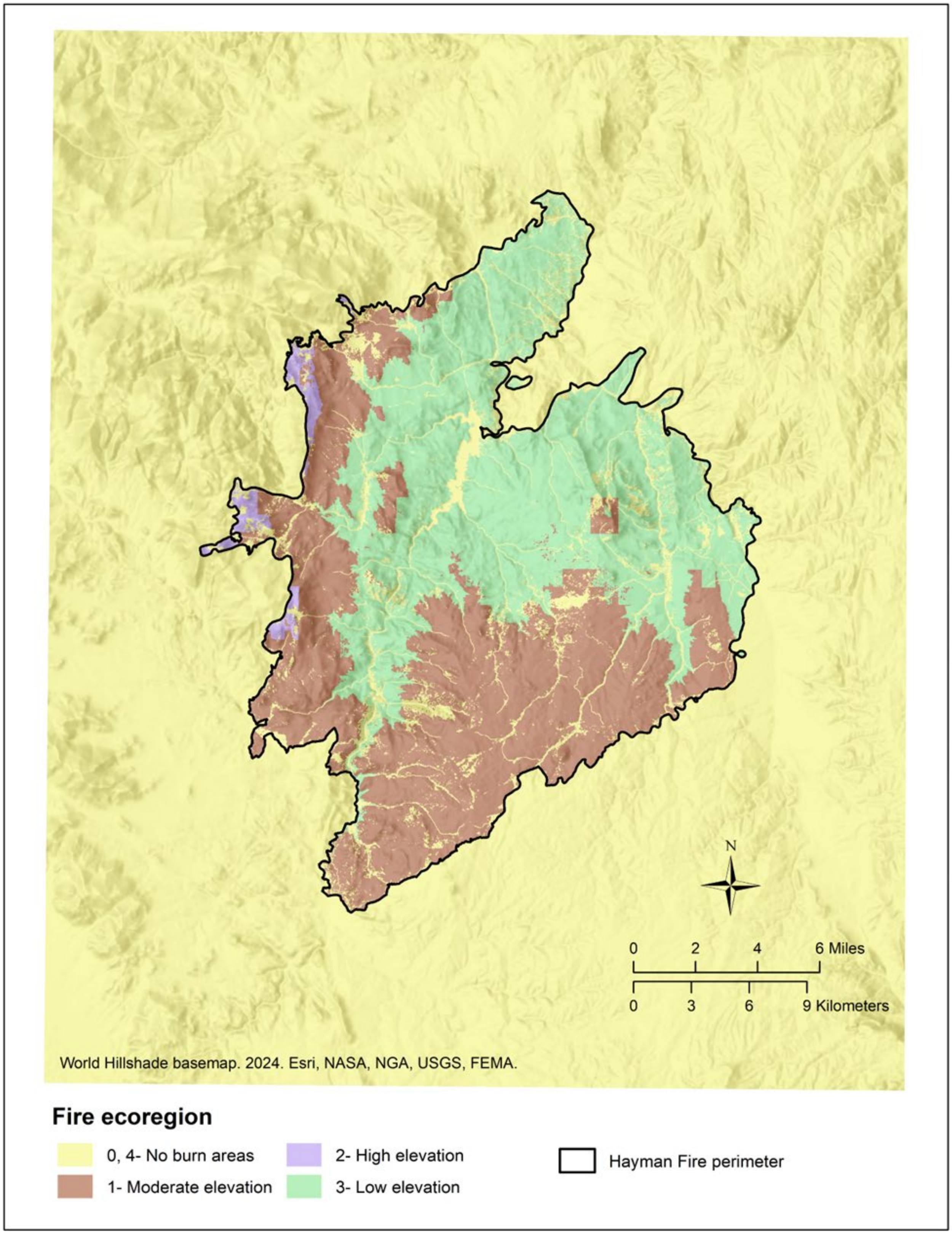

Figure B.1. Map of the fire ecoregions used in the LANDIS-II Hayman Fire simulation.

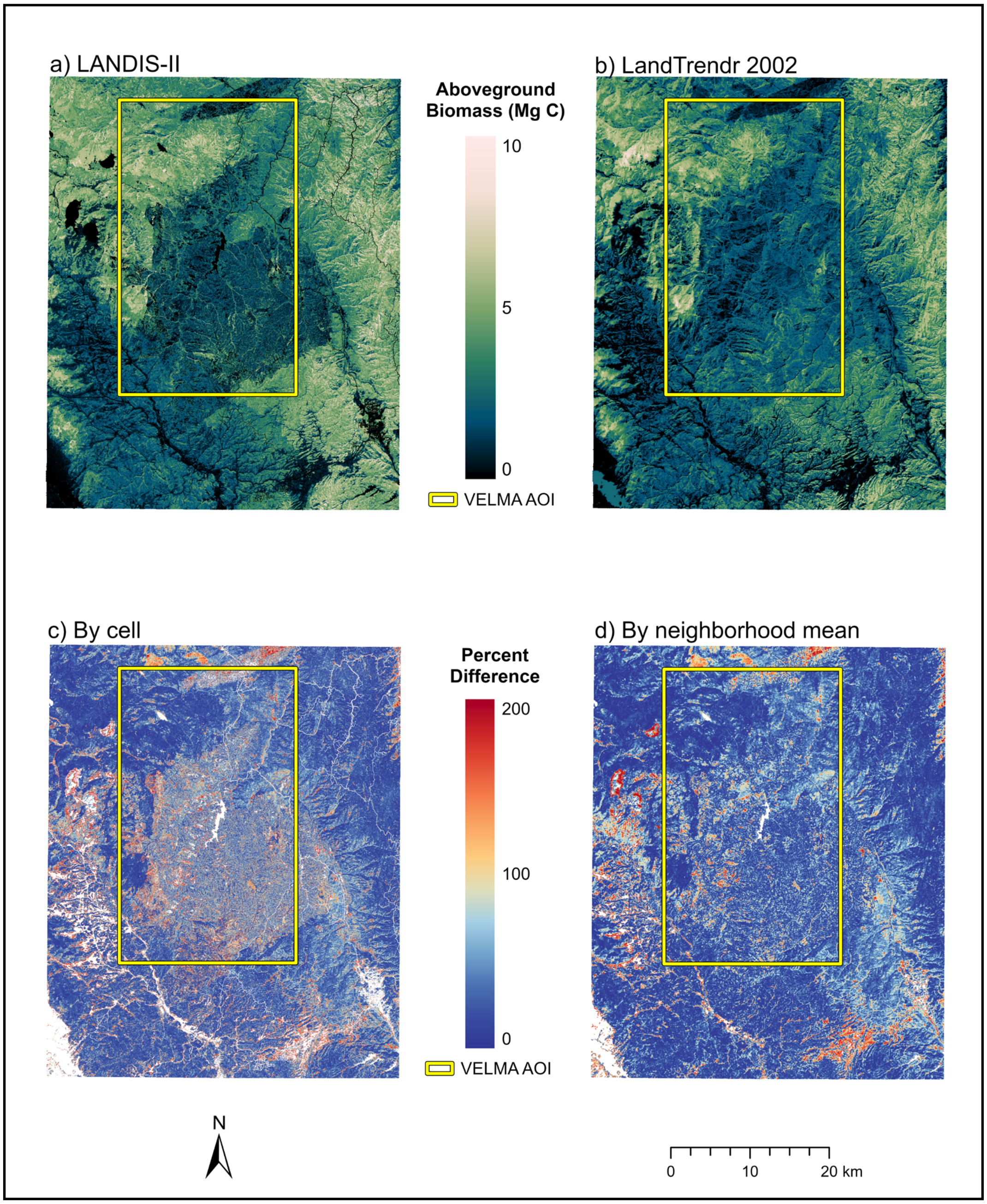

Figure B.2. Top, the aboveground biomass in megagrams of carbon as depicted by a) the LANDIS-II simulation of the 2002 Hayman Fire and b) the 2002 band of the LandTrendr Biomass, CONUS (1990-2017) dataset (Hooper and Kennedy, 2018). The yellow rectangle denotes the VELMA area of interest. Bottom, the percentage difference in aboveground biomass between these two layers is shown in c) as a direct cell-by-cell value comparison. While in d) mean biomass values for each pixel were calculated using a 3x3 cell neighborhood (about 0.81 ha) prior to calculating the percentage difference. Nodata pixels were ignored during mean calculations. Pixels with zero biomass values were nulled and ignored in percentage difference calculations.

References

Colorado State Forest Service. (n.d.). *Colorado's major tree species*. Colorado State University. https://csfs.colostate.edu/colorado-trees/colorados-major-tree-species/

Creutzburg, M. K., Scheller, R. M., Lucash, M. S., LeDuc, S. D., & Johnson, M. G. (2017). Forest management scenarios in a changing climate: trade-offs between carbon, timber, and old forest. *Ecological Applications*, 27(2), 503-518. https://doi.org/10.1002/eap.1460

Geospatial Multi-Agency Coordination Group. (n.d.). *US_HIST_FIRE_PERIM_2002_DD83* [Dataset]. National Interagency Fire Center. https://data-nifc.opendata.arcgis.com/

Hooper, S., & Kennedy, R. E. (2018). A spatial ensemble approach for broad-area mapping of land surface properties. *Remote Sensing of Environment*, *210*, 473 - 489. https://doi.org/10.1016/j.rse.2018.03.032

Jung, C. G., Keyser, A. R., Remy, C. C., Krofcheck, D., Allen, C. D., & Hurteau, M. D. (2023). Topographic information improves simulated patterns of post-fire conifer regeneration in the southwest United States. *Global Change Biology*, *29*(15), 4342-4353. https://doi.org/10.1111/gcb.16764

Loudermilk, L. Southern Research Station, Center for Forest Disturbance Science, U.S. Forest Service, Athens, GA. Personal communication, November 18, 2020.

Moench, R. (2006). *Vegetative recovery after wildfire*. Natural Resources Series|Forestry no. 6.307. Colorado State University Cooperative Extension & Colorado State Forest Service. https://static.colostate.edu/client-files/csfs/pdfs/06307.pdf

Scheller, R. M. (2016a, September 19). *LANDIS-II: Project-Sierra-Nevada-2007 (FIRE-Low.txt)*. GitHub. https://github.com/LANDIS-II-Foundation/Project-Sierra-Nevada-2007/blob/master/LandisRuns/FIRE-Low.txt

Scheller, R. M. (2016b, September 19). *LANDIS-II: Project-Sierra-Nevada-2007 (FUELS-Biomass.txt)*. GitHub. https://github.com/LANDIS-II-Foundation/Project-Sierra-Nevada-2007/blob/master/LandisRuns/FUELS-Biomass.txt

Scheller, R. M., & Cassell, B. A. (2019a, April 4). *LANDIS-II: Project-Malheur-Fuel-Treatment (DynamicBiomassFuels_Input.txt)*. GitHub. https://github.com/LANDIS-II-Foundation/Project-Malheur-Fuel-Treatment/blob/master/Climate_Change_Manuscript_Supplemental%20_Files/LANDIS-II%20Input%20Files/DynamicBiomassFuels_Input.txt

Scheller, R. M., & Cassell, B. A. (2019b, April 4). *LANDIS-II: Project-Malheur-Fuel-Treatment (DynamicFire_Input.txt)*. GitHub. https://github.com/LANDIS-II-Foundation/Project-Malheur-Fuel-Treatment/blob/master/Climate_Change_Manuscript_Supplemental%20_Files/LANDIS-II%20Input%20Files/DynamicFire_Input.txt

Scheller, R. M., & Cassell, B. A. (2019c, April 4). *LANDIS-II: Project-Malheur-Fuel-Treatment (FireRegion_Input.txt)*. GitHub. https://github.com/LANDIS-II-Foundation/Project-Malheur-Fuel-

Treatment/blob/master/Climate_Change_Manuscript_Supplemental%20_Files/LANDIS-II%20Input%20Files/FireRegion_Input.txt

Scheller, R. M., & Kretchun, A. (2017a, June 26). *LANDIS-II: Project-Lake-Tahoe-Basin-2010 (DynamicFire-base-lower-ign.txt)*. https://github.com/LANDIS-II-Foundation/Project-Lake-Tahoe-Basin-2010/blob/master/Model_Inputs_CJFR/DynamicFire-base-lower-ign.txt

Scheller, R. M., & Kretchun, A. (2017b, June 26). *LANDIS-II: Project-Lake-Tahoe-Basin-2010 (DynamicFuels.txt)*. https://github.com/LANDIS-II-Foundation/Project-Lake-Tahoe-Basin-2010/blob/master/Model_Inputs_CJFR/DynamicFuels.txt

Scheller, R. M., & McCauley, L. (2020a, January 25). *LANDIS-II: Project-Arizona-4FRI (DynamicFire.txt)*. GitHub. https://github.com/LANDIS-II-Foundation/Project-Arizona-4FRI/blob/master/Input_files/DynamicFire.txt

Scheller, R. M., & McCauley, L. (2020b, January 25). *LANDIS-II: Project-Arizona-4FRI (DynamicFuels.txt)*. GitHub. https://github.com/LANDIS-II-Foundation/Project-Arizona-4FRI/blob/master/Input_files/DynamicFuels.txt

Sturtevant, B. R., Scheller, R. M., Miranda, B. R., Shinneman, D., & Syphard, A. (2009). Simulating dynamic and mixed-severity fire regimes: A process-based fire extension for LANDIS-II. *Ecological Modelling*, *220*(23), 3380-3393. https://doi.org/10.1016/j.ecolmodel.2009.07.030

United States Department of Agriculture, Forest Service. (2022). *National USFS Fire Perimeter* [Dataset]. https://data.fs.usda.gov/geodata/edw/datasets.php

Vukomanovic, J. College of Natural Resources, North Carolina State University, Raleigh, NC. Personal communication, April 14, 2020.

Western Regional Climate Center. (2020, November 13). RAWS USA Climate Archive [Dataset]. https://raws.dri.edu/
