# Supplementary material for: Model linkage to assess forest disturbance impacts on water quality: A wildfire case study using LANDIS(II)-VELMA
Source: Environ Model Softw. Author manuscript; Available in PMC 2025 Sep 1. (PMC11457591; doi:10.1016/j.envsoft.2024.106134)
Supplement: Supplement3 [file NIHMS2016776-supplement-Supplement3.pdf]

Journal: Environmental Modelling & Software

Title: Model linkage to assess forest disturbance impacts on water quality: a wildfire case study using LANDIS(II)-VELMA

Authors: Kar'retta Venable, John M. Johnston, Stephen D. LeDuc, and Lourdes Prieto

Appendix A: LANDIS-II succession extension input parameters

This appendix covers the ecoregions, initial forest communities, and input parameters values used to run LANDIS-II (version 7.0) (The LANDIS-II Foundation, 2018b) with the Biomass Succession extension (version 6.0) (Scheller and Mladenoff, 2004) in the Colorado Front Rage mountains. Specifically, Tables A.1 - A.7 contain the values used during the calibration of the aboveground live biomass of the initial (succession time step zero) forest communities raster (discussed later). As the tables indicate, values were compiled from the literature, online sources, and personal communication. For more information on these parameters, please consult the model user guide (http://www.landis-ii.org/install) and the extension user guide (http://www.landis-ii.org/extensions).

The ecoregions (Fig. A.1) were developed using an iso cluster unsupervised classification technique (Syphard et al., 2011) in ArcGIS (Esri, 2020) based on characteristics that greatly influenced our location: elevation (30m, United States Department of Agriculture, Natural Resources Conservation Service, National Geospatial Center of Excellence [USDA NRCS NGCE], 2019), slope (derived from elevation), temperature (1981-2010 PRISM derived maximum and minimum averages, USDA NRCS NGCE, 2012), and precipitation (1981-2010 PRISM derived annual average, USDA NRCS NGCE, 2012). The resulting ecoregions roughly approximated the foothills, montane, and subalpine Colorado life zones (Addington et al., 2018; Kaufmann et al., 2006; Ramaley, 1907), with the montane zone subdivided into dry and mesic regions. The alpine ecoregion was added by masking the cells that corresponded to about 11,500 feet or higher in the elevation raster. Then, cells that corresponded to open water, developed land, barren land, or hay/pasture per the 2001 National Land Cover Database (NLCD) raster (Dewitz & US Geological Survey [USGS], 2019) were classified as inactive.

The climate input files (Lucash and Scheller, 2019) were created using Daymet 1 km daily surface weather data (Thornton et al., 2016) downloaded by ecoregion from the USGS Geo Data Portal (USGS, 2020) using an area weighted algorithm. For the model's spin-up phase, we used 1980-2001 weather data with the daily random year option. For the simulation years, we used 2000-2002 weather data with the daily sequenced years option.

Below is a description of the creation and calibration of the initial forest communities.

Creation of initial forest communities' raster

To run LANDIS-II, the user must provide a forest communities map to represent the initial conditions in the area to be modeled. In this map, trees are grouped by species (or functional groups) and age cohorts (The LANDIS-II Foundation, 2018a). For this study, the goal was to create a map that resembled the forest communities that existed prior to the 2002 Colorado Hayman Fire. To create a preliminary version of the initial forest communities map, we used the LANDIS Species Map Creator tool from Landscape Builder version 2.1.5 (Dijak, n.d.). It produced a 30-meter raster where pixel values represented map codes and a text file that identified the tree species and age cohorts per map code.

Following is a summary of how we populated Landscape Builder. For details on tool inputs, please refer to Dijak's 2013 paper. We obtained Forest Inventory and Analysis (FIA) data (USDA, Forest Service [USDA FS], 2024) from the FIA Data Mart (USDA FS, Northern Research Station, 2019/2020). Data from the 2002-2005 inventories were used to identify the 11 most common tree species in the study area and to determine their oldest ages. These ages were substituted for the species maximum longevity to create more representative communities. Species-specific diameter-age regression equations were developed using diameter-age paired records from FIA. Data from the 1984 and 2002-2005 inventories was used when at least 50 paired records per species were available. Otherwise, data from all Colorado and all inventory years on record (1984, 2002-2018) was used. The landform raster was created using topography tools (Dilts, 2019), an elevation raster (30m, USDA NRCS NGCE, 2019), and hydrography layers (US Environmental Protection Agency [USEPA] and US Geological Survey [USGS], 2012). FIA physiographic class codes and slope-aspect ranges were associated with the landforms. The land cover map was derived from the 2001 LANDFIRE existing vegetation type layer (LANDFIRE, 2001). FIA forest type codes were associated with the vegetation classes. The FIA unit map was created using county and survey unit information. Stand age size classes were defined and the species that could stump sprout were identified. After Landscape Builder created the forest communities map, the shrubs were added according to the Shrub/Scrub category from the 2001 NLCD raster (Dewitz & USGS, 2019).

Calibration of initial forest communities' raster

The preliminary version of the forest communities map was run in LANDIS-II to visualize the initial (succession time step zero) distribution of tree species and shrubs and to obtain the initial simulated aboveground biomass, which is calculated during the model's spin-up phase (Scheller and

Miranda, 2020). When deemed appropriate, adjustments were made to the location of tree species including converting some shrubs to Gambel oak or Rocky Mountain juniper. Several sources (Colorado State Forest Service, n.d.-a; LANDFIRE, 2001; USDA FS, n.d.; USDA FS, 2020) were consulted during this process.

When calibrating the simulated aboveground live biomass of the forest communities at succession time step zero, the goal was for LANDIS-II to produce an initial total aboveground biomass that was comparable (within 5%) to the biomass estimate derived from the National Biomass and Carbon Dataset (NBCD 2000) (Kellndorfer et al., 2007-2009, 2013). Biomass by ecoregion comparisons were also performed. For this purpose, it was necessary to combine the alpine and subalpine ecoregions as discrepancies in the location of the alpine area shifted some tree biomass above the tree line zone. It was also necessary to merge the inactive ecoregion into its neighboring ecoregions to account for discrepancies in the location of the inactive areas. Calibration was accomplished by doing iterative adjustments to several model inputs: the map codes (species composition and age cohorts), the maximum aboveground net primary productivity per species-ecoregion combination, the maximum biomass per species-ecoregion combination, and the spin-up mortality fraction. The total aboveground live biomass of the calibrated initial forest communities was 0.02 % different from the NBCD 2000 aboveground live biomass. It was 0.53% different when compared to the year 2000 band of the LandTrendr (LTr) Biomass, CONUS (1990-2017) dataset (Hooper and Kennedy, 2018). The final biomass by ecoregion percentage differences with the NBCD 2000 were equal or less than 0.29%. A depiction of the aboveground biomass in the study areas can be seen in Fig. A.2.

Tables A.1 - A.7. Superscript numbers indicate the sources consulted to determine parameter value.

Table A.1. Life history parameters for tree species and functional group used in LANDIS-II simulation.

**Common name**	**Species name**	**Species code**	**Longevity** (years) [2, 8-9, 11, 13-14, 17, 23, 26-28]	**Sexual maturity** (years) [8, 10, 13-14, 17, 20, 23-24, 27-28]	**Shade tolerance** (1-5) [8-9, 11, 14-17, 20, 24, 27-28]	**Fire tolerance** (1-5) [2, 8-9, 11, 17, 20, 23-28]	**Seed dispersal distance** (meters)		**Vegetative reproduction probability** [1-2, 8-9, 11, 13-14, 17, 23, 26-28]	**Resprouting age** (years) [2, 8-9, 11, 13-14, 17, 23, 26-28]		**Post-fire regeneration** [2, 4, 8-9, 11, 13-14, 17, 23-24, 26-28]
							**Effective distance** [2-3, 5-6, 8, 11, 14, 19, 21-22, 28]	**Maximum distance** [2-3, 7-8, 10-14, 17-19, 22-23, 28]		**Minimum**	**Maximum**	
Subalpine Fir	*Abies lasiocarpa*	abielasi	250	20	5	1	30	60	0	0	0	none
Rocky Mountain Juniper	*Juniperus scopulorum*	juniscop	300	15	2	1	20	500	0	0	0	none
Engelmann Spruce	*Picea engelmannii*	piceenge	600	25	4	1	30	180	0	0	0	none
Blue Spruce	*Picea pungens*	picepung	600	20	3	1	30	90	0	0	0	none
Rocky Mountain Bristlecone Pine	*Pinus aristata*	pinuaris	1600	25	1	2	200	5000	0	0	0	none
Lodgepole Pine	*Pinus contorta*	pinucont	250	5	2	2	30	100	0	0	0	serotiny
Ponderosa Pine	*Pinus ponderosa*	pinupond	400	10	2	5	35	150	0	0	0	none
Limber Pine	*Pinus flexilis*	pinuflex	1000	20	3	3	30	5000	0	0	0	none
Douglas-fir	*Pseudotsuga menziesii*	pseumenz	750	15	3	3	30	1000	0	0	0	none
Quaking Aspen	*Populus tremuloides*	poputrem	150	15	1	2	30	1000	0.9	1	150	resprout
Gambel Oak	*Quercus gambelii*	quergamb	90	10	3	3	30	1000	1	1	80	resprout
shrubs		shrubs	80	5	1	1	30	750	0	0	0	none

For Shade tolerance and Fire tolerance 1 represents the lowest tolerance and 5 the highest.

[1]Addington et al. (2018)
[2]Anderson (2003)
[3]Chambers et al. (1999)
[4]Colorado State Forest Service (n.d.-a)
[5]Coop & Schoettle (2009)
[6]Dawe & Flannigan (2020)
[7]Erickson (2001)
[8]Fryer (2004)
[9]Howard (1996)
[10]Howard (2003)
[11]Hurteau et al. (2016)
[12]Jay's Bird Barn (n.d.)
[13]Johnson (2001)
[14]Liang et al. (2017)
[15]The Morton Arboretum (n.d.)
[16]Noble (1990)
[17]Pavek (1993)
[18]Schaming (n.d.)
[19]Scheller (2016b)
[20]Scheller & Cassell (2019b)
[21]Scheller et al. (2018)
[22]Scheller & McCauley (2020b)
[23]Scher (2002)
[24]Simonin (2000)
[25]Steele (1990)
[26]Uchytil (1991a)
[27]Uchytil (1991b)
[28]Vukomanovic (2020)

Table A.2. LANDIS-II minimum relative biomass by shade class (1-5) per ecoregion table. Shade class 1 represents the least amount of shade while class 5 represents the most.

Shade Class	Ecoregion[1]				
	eco1	eco2	eco3	eco4	eco5
1	5%	5%	5%	5%	5%
2	25%	25%	25%	25%	25%
3	50%	50%	50%	50%	50%
4	80%	80%	80%	80%	80%
5	100%	100%	100%	100%	100%

[1]Kretchun & Scheller (2017b)

Table A.3. LANDIS-II probability of establishment by light condition (0-5) per species shade tolerance class (1-5) table. Shade tolerance 1 represents the lowest shade tolerance and 5 represents the highest tolerance. Light condition 0 represents the highest amount of light while 5 represents the least.

Species Shade Tolerance Class	Probability of Establishment by Light Condition [1-3]					
	0	1	2	3	4	5
1	1	0.5	0.02	0	0	0
2	1	1	0.5	0.02	0	0
3	0.5	1	1	1	0.5	0.1
4	0	0.01	0.6	1	1	0.5
5	0	0	0.01	0.5	1	1

[1]Kretchun & Scheller (2017b)
[2]Scheller (2016a)
[3]Scheller & Creutzburg (2016)

Table A.4. LANDIS-II species biomass parameters table.

Species Code	Leaf Longevity (years) [1, 3-4, 6, 10, 13-14]	Woody (Bole) Decay Rate [2, 4-5, 7-8]	Mortality Curve Shape Parameter [4-5, 7, 10-11]	Growth Curve Shape Parameter [4, 12]	% Leaf Lignin (as a decimal) [3-4, 8-9, 13]
abielasi	8	0.035	10	1	0.25
juniscop	5	0.05	5	1	0.2
piceenge	7.5	0.028	10	1	0.2
picepung	10	0.028	10	1	0.2
pinuaris	10	0.023	10	1	0.2
pinucont	3.5	0.023	10	1	0.25
pinupond	4.5	0.1	10	1	0.24
pinuflex	3	0.1	10	1	0.2
pseumenz	7	0.005	10	1	0.2
poputrem	1	0.045	10	0	0.141
quergamb	1	0.1	15	0	0.175
shrubs	1	0.2	10	0	0.25

[1]Fryer (2004)
[2]Harmon & Fasth (2005) *
[3]Hurteau et al. (2016)
[4]Kretchun & Scheller (2017b)
[5]Lucash & Scheller (2018)
[6]Reich et al. (1999)
[7]Scheller (2016a)
[8]Scheller & Cassell (2019a)
[9]Scheller et al. (2011)
[10]Scheller et al. (2019)
[11]Scheller & Mladenoff (2004)
[12]Tremblay et al. (2018)
[13]Vukomanovic (2020)
[14]Zeleznik (n.d.)

* Acknowledgements for the use of woody decay rates obtained from source #2

"Data [and/or facilities] were provided by the HJ Andrews Experimental Forest and Long Term Ecological Research (LTER) program, administered cooperatively by the USDA Forest Service Pacific Northwest Research Station, Oregon State University, and the Willamette National Forest. This material is based upon work supported by the National Science Foundation under the LTER Grants: LTER8 DEB-2025755 (2020-2026) and LTER7 DEB-1440409 (2012-2020)."

Table A.5. LANDIS-II actual evapotranspiration (AET) by ecoregion table.

Ecoregion	AET [1-3] (mm)
eco1	315
eco2	350
eco3	325
eco4	350
eco5	210

[1]Esri et al. (2020)
[2]Kolka & Wolf (1998)
[3]Sanford & Selnick (2013)

Table A.6. LANDIS-II biomass dynamic inputs table. Values listed for the maximum aboveground net primary productivity and the maximum aboveground biomass reflect adjustments made during calibration. As a result, some maximum aboveground biomass values were outside the ranges seen in the sources consulted. The Probability of Mortality field, not shown, was kept with its default value of zero.

Year	Ecoregion	Species Code	Probability of Establishment [1-6, 8-9, 11, 13, 15, 18-19, 21-25, 27]	Maximum Aboveground Net Primary Productivity (ANPP) (g m^{-2} yr^{-1}) [7, 10, 12, 16, 20, 26]	Maximum Aboveground Biomass (AGB) (g m^{-2}) [10, 12, 14, 17, 26]
0	eco1	abielasi	0	0	0
0	eco1	juniscop	0.3	700	5,000
0	eco1	piceenge	0.25	950	13,500
0	eco1	picepung	0.5	850	12,000
0	eco1	pinuaris	0.45	700	10,000
0	eco1	pinucont	0.4	500	10,000
0	eco1	pinupond	0.65	500	2,000
0	eco1	pinuflex	0.5	850	13,000
0	eco1	pseumenz	0.65	850	12,000
0	eco1	poputrem	0.6	600	7,000
0	eco1	quergamb	0.5	350	2,500
0	eco1	shrubs	0.75	250	767
0	eco2	abielasi	0.6	800	2,000
0	eco2	juniscop	0	0	0
0	eco2	piceenge	0.75	950	40,000
0	eco2	picepung	0.4	850	10,000
0	eco2	pinuaris	0.5	700	12,000
0	eco2	pinucont	0.65	650	12,000
0	eco2	pinupond	0	0	0

Table A.6. LANDIS-II biomass dynamic inputs table, Continued

Year	Ecoregion	Species Code	Probability of Establishment [1-6, 8-9, 11, 13, 15, 18-19, 21-25, 27]	Maximum Aboveground Net Primary Productivity (ANPP) (g m^{-2} yr^{-1}) [7, 10, 12, 16, 20, 26]	Maximum Aboveground Biomass (AGB) (g m^{-2}) [10, 12, 14, 17, 26]
0	eco2	pinuflex	0.65	700	20,000
0	eco2	pseumenz	0.4	900	12,000
0	eco2	poputrem	0.6	600	10,000
0	eco2	quergamb	0	0	0
0	eco2	shrubs	0.5	200	211
0	eco3	abielasi	0	0	0
0	eco3	juniscop	0.65	700	7,000
0	eco3	piceenge	0	0	0
0	eco3	picepung	0.45	850	10,000
0	eco3	pinuaris	0	0	0
0	eco3	pinucont	0.4	650	5,000
0	eco3	pinupond	0.75	900	50,000
0	eco3	pinuflex	0.45	850	10,000
0	eco3	pseumenz	0.65	900	45,000
0	eco3	poputrem	0.5	600	10,000
0	eco3	quergamb	0.6	350	2,500
0	eco3	shrubs	0.75	250	4,045
0	eco4	abielasi	0.25	950	5,000
0	eco4	juniscop	0.25	700	5,500
0	eco4	piceenge	0.45	950	20,000
0	eco4	picepung	0.6	850	12,000
0	eco4	pinuaris	0.4	700	10,000
0	eco4	pinucont	0.65	650	12,000
0	eco4	pinupond	0.7	900	25,000
0	eco4	pinuflex	0.65	950	20,000
0	eco4	pseumenz	0.75	900	50,000
0	eco4	poputrem	0.65	800	11,000
0	eco4	quergamb	0.1	350	2,500
0	eco4	shrubs	0.75	250	1,030
0	eco5	abielasi	0	0	0
0	eco5	juniscop	0	0	0
0	eco5	piceenge	0	0	0
0	eco5	picepung	0	0	0
0	eco5	pinuaris	0	0	0

Table A.6. LANDIS-II biomass dynamic inputs table, Continued

Year	Ecoregion	Species Code	Probability of Establishment [1-6, 8-9, 11, 13, 15, 18-19, 21-25, 27]	Maximum Aboveground Net Primary Productivity (ANPP) (g m^{-2} yr^{-1}) [7, 10, 12, 16, 20, 26]	Maximum Aboveground Biomass (AGB) (g m^{-2}) [10, 12, 14, 17, 26]
0	eco5	pinucont	0	0	0
0	eco5	pinupond	0	0	0
0	eco5	pinuflex	0	0	0
0	eco5	pseumenz	0	0	0
0	eco5	poputrem	0	0	0
0	eco5	quergamb	0	0	0
0	eco5	shrubs	0.5	200	284

[1]Addington et al. (2018)
[2]Anderson (2003)
[3]Colorado State Forest Service (n.d.-a)
[4]Colorado State Forest Service (n.d.-b)
[5]Fryer (2004)
[6]Graham & Jain (2005)
[7]Henne et al. (2021)
[8]Howard (1996)
[9]Howard (2003)
[10]Hurteau et al. (2016)
[11]Johnson (2001)
[12]Kretchun & Scheller (2017a)
[13]LANDFIRE (2001)
[14]Maxwell & Scheller (2020)
[15]Pavek (1993)
[16]Scheller (2016a)
[17]Scheller & McCauley (2020a)
[18]Scher (2002)
[19]Simonin (2000)
[20]Smith & Resh (1999)
[21]Steinberg (2002)
[22]Uchytil (1991a)
[23]Uchytil (1991b)
[24]USDA, Forest Service (n.d.)
[25]USDA, Forest Service (2020)
[26]Vukomanovic (2020)
[27]Weir (2014)

Table A.7. LANDIS-II fire reduction parameters by fire severity class (1-5) table. Class 1 represents the lowest fire severity while 5 represents the highest.

Fire Severity Class	Wood Reduction [1-2] (proportion of dead wood biomass volatilized)	Litter Reduction [1] (proportion of dead litter biomass volatilized)
1	0.05	0.5
2	0.2	0.75
3	0.4	1
4	0.5	1
5	0.8	1

[1]Scheller & Cassell (2019a)
[2]Scheller et al. (2019)

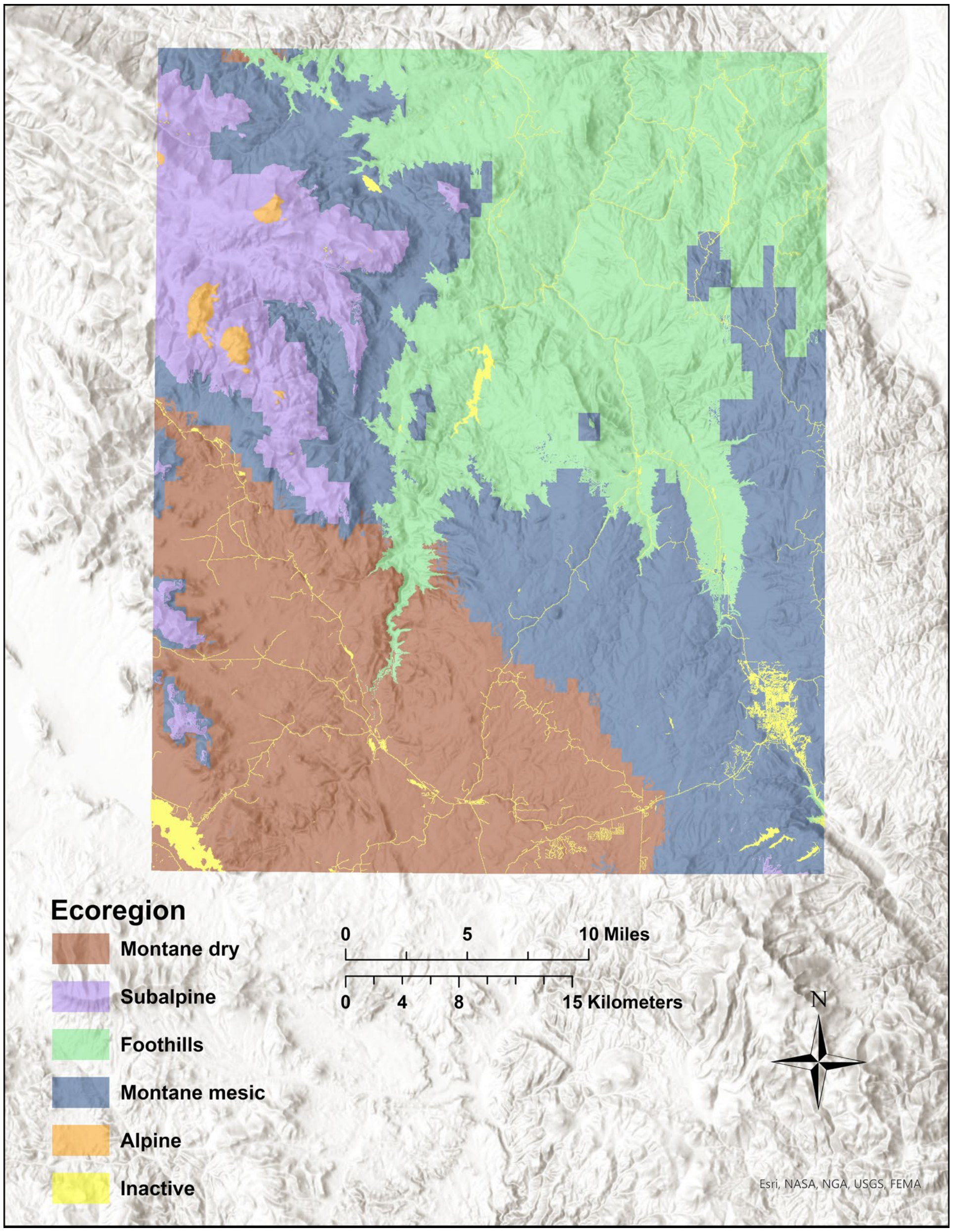

World Hillshade basemap. 2024. Esri, NASA, NGA, USGS, FEMA.

	Approximate life zone	Elevation range		Elevation means and standard deviation	
		Feet	Meters	Feet	Meters
Ecoregion 1	Montane dry	7,801 - 10,000	2,378 - 3,048	8,746 ± 361	2,666 ± 110
Ecoregion 2	Subalpine	8,993 - 11,499	2,741 - 3,505	10,395 ± 517	3,168 ± 158
Ecoregion 3	Foothills	6,100 - 9,827	1,859 - 2,995	7,558 ± 493	2,304 ± 150
Ecoregion 4	Montane mesic	7,696 - 10,902	2,346 - 3,323	8,761 ± 453	2,670 ± 138
Ecoregion 5	Alpine	11,499 - 12,425	3,505 - 3,787	11,761 ± 202	3,585 ± 62
Ecoregion 6	Inactive	-	-	-	-

Figure A.1. Map of the ecoregions used in the LANDIS-II simulation. Elevation ranges and means provided as reference.

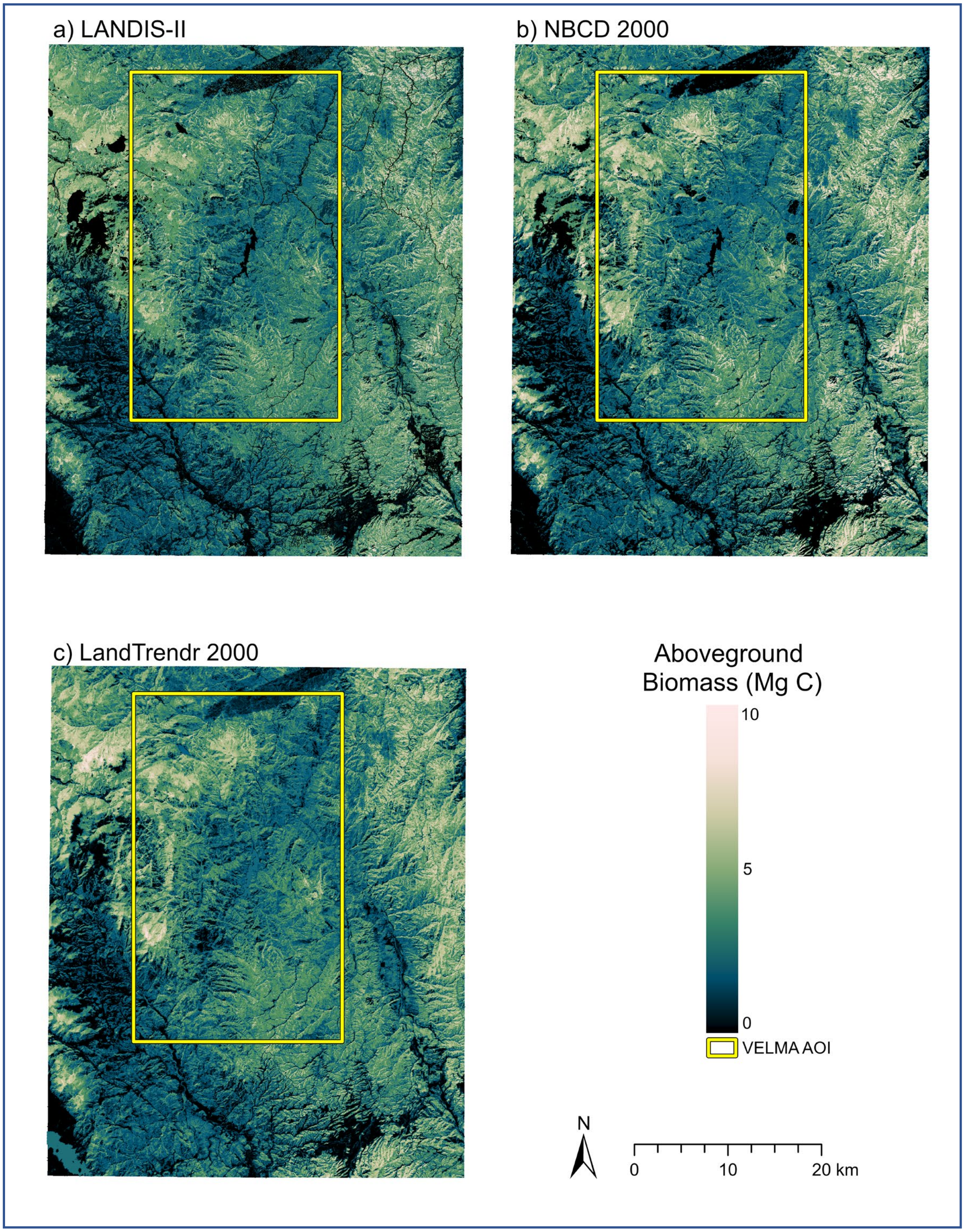

Figure A.2. Aboveground biomass in megagrams of carbon as depicted by a) LANDIS-II simulation at succession time step zero, b) the National Biomass and Carbon Dataset (NBCD 2000) (Kellndorfer et al., 2007-2009, 2013), and c) the LandTrendr Biomass, CONUS (1990-2017) dataset (Hooper and Kennedy, 2018), 2000 band. The yellow rectangle denotes the VELMA area of interest.

References

Addington, R. N., Aplet, G. H., Battaglia, M. A., Briggs, J. S., Brown, P. M., Cheng, A. S., Dickinson, Y., Feinstein, J. A., Pelz, K. A., Regan, C. M., Thinnes, J., Truex, R., Fornwalt, P. J., Gannon, B., Julian, C. W., Underhill, J. L., & Wolk, B. (2018). *Principles and practices for the restoration of ponderosa pine and dry mixed-conifer forests of the Colorado Front Range* (Gen. Tech. Rep. RMRS-GTR-373). United States Department of Agriculture, Forest Service, Rocky Mountain Research Station. https://doi.org/10.2737/RMRS-GTR-373

Anderson, M. D. (2003). *Pinus contorta* var*. latifolia*. In *Fire Effects Information System*, [Online]. United States Department of Agriculture, Forest Service, Rocky Mountain Research Station, Fire Sciences Laboratory. https://www.fs.usda.gov/database/feis/plants/tree/pinconl/all.html

Chambers, J., Vander Wall, S. B., & Schupp, E. (1999). Seed and seedling ecology of piñon and juniper species in the pygmy woodlands of western North America. *Botanical Review*, *65*(1), 1–38. http://www.jstor.org/stable/4354335

Colorado State Forest Service. (n.d.-a). *Colorado's major tree species*. Colorado State University. https://csfs.colostate.edu/colorado-trees/colorados-major-tree-species/

Colorado State Forest Service. (n.d.-b). *Hardwood – Primarily aspen*. Colorado State University. https://csfs.colostate.edu/colorado-forests/forest-types/aspen/

Coop, J., & Schoettle, A. (2009). Regeneration of Rocky Mountain bristlecone pine (*Pinus aristata*) and limber pine (*Pinus flexilis*) three decades after stand-replacing fires. *Forest Ecology and Management.*, *257*, 893–903. https://doi.org/10.1016/j.foreco.2008.10.034

Dawe, D. A., Peters, V. S., & Flannigan, M. D. (2020). Post-fire regeneration of endangered limber pine (*Pinus flexilis*) at the northern extent of its range. *Forest Ecology and Management.*, *457*. https://doi.org/10.1016/j.foreco.2019.117725

Dewitz, J., & United States Geological Survey. (2019). *National Land Cover Database (NLCD) 2019 products (ver. January 2019)* [Data set]. United States Geological Survey. https://doi.org/10.5066/P9KZCM54

Dijak, W. (n.d.). *Landscape Builder* (Version 2.1.5) [Computer software]. United States Department of Agriculture, Forest Service, Northern Research Station. https://www.fs.usda.gov/nrs/software/landscapebuilder/

Dijak, W. (2013). Landscape Builder: Software for the creation of initial landscapes for LANDIS from FIA data. *Computational Ecology and Software*, *3*, 17 - 25. https://research.fs.usda.gov/treesearch/43997

Dilts, T. E. (2019). *Topography tools for ArcGIS 10.3 and earlier*. [Computer software]. University of Nevada Reno. https://www.arcgis.com/home/item.html?id=b13b3b40fa3c43d4a23a1a09c5fe96b9

Erickson, L. (2001, January 5). *For the birds radio program: Bohemian waxwings.* Laura Erickson's for the birds. https://www.lauraerickson.com/radio/program/11266/bohemian-waxwings/

Esri. (2020). ArcGIS Pro (Version 2.4) [Computer software]. https://www.esri.com/en-us/arcgis/products/arcgis-pro/overview

Esri, University of Montana, & National Aeronautics and Space Administration. (2020). *Average annual actual evapotranspiration in mm/year* [Data set]. https://www.arcgis.com/home/item.html?id=31f7c3727abf42249a43fe8f25470af4

Fryer, J. L. (2004). *Pinus aristata*. In *Fire Effects Information System*, [Online]. United States Department of Agriculture, Forest Service, Rocky Mountain Research Station, Fire Sciences Laboratory. https://www.fs.usda.gov/database/feis/plants/tree/pinari/all.html

Graham, R. T., & Jain, T. B. (2005). Ponderosa pine ecosystems. In M. W. Ritchie, D. A. Maguire, & A. Youngblood (Tech. coordinators), *Proceedings of the symposium on ponderosa pine: Issues, trends, and management*. 2004 October 18-21, Klamath Falls, OR (Gen. Tech. Rep. PSW-GTR-198) (pp. 1 - 32). United States Department of Agriculture, Forest Service, Pacific Southwest Research Station. https://www.fs.usda.gov/psw/publications/documents/psw_gtr198/psw_gtr198_a.pdf

Harmon, M., & Fasth B. (2005). *Bole decomposition rates of seventeen tree species in western U.S.A*. (Publication 4952). Oregon State University. https://andrewsforest.oregonstate.edu/sites/default/files/lter/pubs/webdocs/reports/decomp/cwd_decomp_web.htm

Henne, P. D., Hawbaker, T. J., Scheller, R. M., Zhao, F., He, H. S., Xu, W., & Zhu, Z. (2021). Increased burning in a warming climate reduces carbon uptake in the Greater Yellowstone Ecosystem despite productivity gains. *Journal of Ecology*, 109(3), 1148-1169. https://doi.org/10.1111/1365-2745.13559

Hooper, S., & Kennedy, R. E. (2018). A spatial ensemble approach for broad-area mapping of land surface properties. *Remote Sensing of Environment*, *210*, 473 - 489. https://doi.org/10.1016/j.rse.2018.03.032

Howard, J. L. (1996). *Populus tremuloides*. In *Fire Effects Information System*, [Online]. United States Department of Agriculture, Forest Service, Rocky Mountain Research Station, Fire Sciences Laboratory. https://www.fs.usda.gov/database/feis/plants/tree/poptre/all.html

Howard, J. L. (2003). *Pinus ponderosa* var. *brachyptera, P. p.* var. *scopulorum.* In *Fire Effects Information System*, [Online]. United States Department of Agriculture, Forest Service, Rocky Mountain Research Station, Fire Sciences Laboratory. https://www.fs.usda.gov/database/feis/plants/tree/pinpons/all.html

Hurteau, M. D., Liang, S., Martin, K. L., North, M. P., Koch, G. W., & Hungate, B. A. (2016). Restoring forest structure and process stabilizes forest carbon in wildfire-prone southwestern ponderosa pine forests. *Ecological Applications*, *26*(2), 382–391. https://doi.org/10.1890/15-0337

Jay's Bird Barn. (n.d.). *How fast do birds fly?* https://www.jaysbirdbarn.com/fast-birds-fly/

Johnson, K. A. (2001). *Pinus flexilis*. In *Fire Effects Information System*, [Online]. United States Department of Agriculture, Forest Service, Rocky Mountain Research Station, Fire Sciences Laboratory. https://www.fs.usda.gov/database/feis/plants/tree/pinfle/all.html

Kaufmann, M. R., Veblen, T. T., & Romme, W. H. (2006). *Historical fire regimes in ponderosa pine forests of the Colorado Front Range, and recommendations for ecological restoration and fuels management*. Front Range Fuels Treatment Partnership roundtable: Findings of the Ecology Workgroup. Front Range Fuels Treatment Partnership. https://research.fs.usda.gov/treesearch/61131

Kellndorfer, J., Walker, W., Kirsch, K., Fiske, G., Bishop, J., Lapoint, L., Hoppus, M., & Westfall, J. (2007-2009). *The National Biomass and Carbon Dataset 2000 (NBCD 2000)*. The Woods Hole Research Center, Falmouth, MA.

Kellndorfer, J., Walker, W., Kirsch, K., Fiske, G., Bishop, J., Lapoint, L., Hoppus, M., & Westfall, J. (2013). *NACP aboveground biomass and carbon baseline data, V.2 (NBCD 2000), U.S.A., 2000* [Dataset]. ORNL DAAC. https://doi.org/10.3334/ORNLDAAC/1161

Kolka, R. K., & Wolf, A. T. (1998). *Estimating actual evapotranspiration for forested sites: Modifications to the Thornthwaite model*. (Research Note SRS-6). USDA Forest Service, Southern Research Station, Center for Forested Wetlands Research, Savannah River Ecology Lab. https://www.srs.fs.usda.gov/pubs/rn/rn_srs006.pdf

Kretchun, A., & Scheller, R. M. (2017a, June 1). *LANDIS-II: Project-Idaho-Aspen (biomass-succession-dynamic-inputs_RCEW.txt)*. GitHub. https://github.com/LANDIS-II-Foundation/Project-Idaho-Aspen/blob/master/RCEW_inputs/biomass-succession-dynamic-inputs_RCEW.txt

Kretchun, A., & Scheller, R. M. (2017b, June 1). *LANDIS-II: Project-Idaho-Aspen (biomass-succession_RCEW.txt)*. GitHub. https://github.com/LANDIS-II-Foundation/Project-Idaho-Aspen/blob/master/RCEW_inputs/biomass-succession_RCEW.txt

LANDFIRE. (2001). *Existing vegetation type layer, LANDFIRE v. 1.0.5* [Dataset]. United States Department of the Interior, Geological Survey. https://www.landfire.gov/viewer/

The LANDIS-II Foundation. (2018a). *Forecasting forested landscapes: An introduction to LANDIS-II with exercises* (5th ed.). CreateSpace Independent Publishing Platform.

The LANDIS-II Foundation. (2018b). *LANDIS-II model* (Version 7.0) [Computer software]. http://www.landis-ii.org/install

Liang, S., Hurteau, M. D., & Westerling, A. L. (2017). Response of Sierra Nevada forests to projected climate–wildfire interactions. *Global Change Biology*, *23*(5), 2016–2030. https://doi.org/10.1111/gcb.13544

Lucash, M. & Scheller, R. M. (2018, August 20). *LANDIS-II: Project-Menominee (NECN_Succession_092017.txt)*. GitHub. https://github.com/LANDIS-II-Foundation/Project-

Menominee-2016/blob/master/iVR_paper_Huang_etal_2018/LANDIS_Sim_Inputs/NECN_Succession_092017.txt

Lucash, M. S. & Scheller, R. M. (2019). *LANDIS-II Climate Library v4.0 user guide*. The LANDIS-II Foundation. https://github.com/LANDIS-II-Foundation/Library-Climate/blob/master/docs/LANDIS-II%20Climate%20Library%20v4.0%20User%20Guide.pdf

Maxwell, C., & Scheller, R. M. (2020, August 21). *LANDIS-II: Project-Tahoe-Central-Sierra-2019 (NECN_Succession.txt)*. GitHub. https://github.com/LANDIS-II-Foundation/Project-Tahoe-Central-Sierra-2019/blob/master/LANDIS%20Inputs/NECN_Succession.txt

The Morton Arboretum. (n.d.). *Limber pine*. https://www.mortonarb.org/trees-plants/tree-plant-descriptions/limber-pine

Noble, D. L. (1990). *Rocky Mountain juniper*. In R. M. Burns & B. H. Honkala (tech. coords.). *Silvics of North America*: *1. Conifers*. (Agriculture Handbook 654 [Vol. 2]). United States Department of Agriculture, Forest Service. https://www.srs.fs.usda.gov/pubs/misc/ag_654/volume_1/juniperus/scopulorum.htm

Pavek, D. S. (1993). *Picea pungens*. In *Fire Effects Information System*, [Online]. United States Department of Agriculture, Forest Service, Rocky Mountain Research Station, Fire Sciences Laboratory. https://www.fs.usda.gov/database/feis/plants/tree/picpun/all.html

Ramaley, F. (1907). Plant zones in the Rocky Mountains of Colorado. *Science*. 26(671), 642–643. https://www.jstor.org/stable/1631836

Reich, P. B., Ellsworth, D. S., Walters, M. B., Vose, J. M., Gresham, C., Volin, J. C., & Bowman, W. D. (1999). Generality of leaf trait relationships: A test across six biomes. *Ecology*, *80*(6), 1955–1969. https://doi.org/10.1890/0012-9658(1999)080[1955:GOLTRA]2.0.CO;2

Sanford, W. E., & Selnick, D. L. (2013). Estimation of evapotranspiration across the conterminous United States using a regression with climate and land-cover data. *JAWRA Journal of the American Water Resources Association*, *49*(1), 217–230. https://doi.org/10.1111/jawr.12010

Schaming, T. (n.d.). *Learn the secrets of Clark's nutcrackers*. Cornell University Crowdfunding. https://crowdfunding.cornell.edu/project/867

Scheller, R. M. (2016a, September 19). *LANDIS-II: Project-Sierra-Nevada-2007 (biosuccession_v2.txt)*. GitHub. https://github.com/LANDIS-II-Foundation/Project-Sierra-Nevada-2007/blob/master/LandisRuns/biosuccession_v2.txt

Scheller, R. M. (2016b, September 19). *LANDIS-II: Project-Sierra-Nevada-2007 (species.txt)*. GitHub. https://github.com/LANDIS-II-Foundation/Project-Sierra-Nevada-2007/blob/master/LandisRuns/species.txt

Scheller, R. M., & Cassell, B. A. (2019a, April 4). *LANDIS-II: Project-Malheur-Fuel-Treatment (necn-succession_20170601.txt)*. GitHub. https://github.com/LANDIS-II-Foundation/Project-Malheur-

Fuel-Treatment/blob/master/Climate_Change_Manuscript_Supplemental%20_Files/LANDIS-II%20Input%20Files/necn-succession_20170601.txt

Scheller, R. M., & Cassell, B. A. (2019b, April 4). *LANDIS-II: Project-Malheur-Fuel-Treatment (species.txt)*. GitHub. https://github.com/LANDIS-II-Foundation/Project-Malheur-Fuel-Treatment/blob/master/Climate_Change_Manuscript_Supplemental%20_Files/LANDIS-II%20Input%20Files/species.txt

Scheller, R. M., & Creutzburg, M. (2016, October 17). *LANDIS-II: Project-Oregon-Coast-Range (CR_Century_Historic.txt)*. GitHub. https://github.com/LANDIS-II-Foundation/Project-Oregon-Coast-Range/blob/master/Input/LANDIS_Input_Files/CR_Century_Historic.txt

Scheller, R. M., Hua, D., Bolstad, P. V., Birdsey, R. A., & Mladenoff, D. J. (2011). The effects of forest harvest intensity in combination with wind disturbance on carbon dynamics in lake states mesic forests. *Elsevier Ecological Modelling*, *222*(1), 144–153. https://doi.org/10.1016/j.ecolmodel.2010.09.009

Scheller, R. M., Maxwell, C., & Kretchun, A. (2018, November 21). *LANDIS-II: Project-Lake-Tahoe-2017 (species.txt)*. GitHub. https://github.com/LANDIS-II-Foundation/Project-Lake-Tahoe-2017/blob/master/LTW_LANDIS_Scenarios/species.txt

Scheller, R. M., Maxwell, C., & Kretchun, A. (2019, March 20). *LANDIS-II: Project-Lake-Tahoe-2017 (NECN_Succession.txt)*. GitHub. https://github.com/LANDIS-II-Foundation/Project-Lake-Tahoe-2017/blob/master/LTW_LANDIS_Scenarios/NECN_Succession.txt

Scheller, R. M., & McCauley, L. (2020a, January 25). *LANDIS-II: Project-Arizona-4FRI (NECN-successionIPSLCM5ALR.txt)*. GitHub. https://github.com/LANDIS-II-Foundation/Project-Arizona-4FRI/blob/master/Input_files/NECN-successionIPSLCM5ALR.txt

Scheller, R. M., & McCauley, L. (2020b, January 25). *LANDIS-II: Project-Arizona-4FRI (species.txt)*. GitHub. https://github.com/LANDIS-II-Foundation/Project-Arizona-4FRI/blob/master/Input_files/species.txt

Scheller, R. M., & Miranda, B. (2020). *LANDIS-II Biomass Succession v5.2 extension user guide*. The LANDIS-II Foundation. https://github.com/LANDIS-II-Foundation/Extension-Biomass-Succession/tree/master/docs

Scheller, R. M., & Mladenoff, D. J. (2004). A forest growth and biomass module for a landscape simulation model, LANDIS: Design, validation, and application. *Elsevier Ecological Modelling*, *180*(1), 211–229. https://doi.org/10.1016/j.ecolmodel.2004.01.022

Scher, J. S. (2002). *Juniperus scopulorum*. In *Fire Effects Information System*, [Online]. [Forestry]. United States Department of Agriculture, Forest Service, Rocky Mountain Research Station, Fire Sciences Laboratory. https://www.fs.usda.gov/database/feis/plants/tree/junsco/all.html

Simonin, K. A. (2000). *Quercus gambelii*. In *Fire Effects Information* System, [Online]. United States Department of Agriculture, Forest Service, Rocky Mountain Research Station, Fire Sciences Laboratory. https://www.fs.usda.gov/database/feis/plants/tree/quegam/all.html

Smith, F. W., & Resh, S. C. (1999). Age-related changes in production and below-ground carbon allocation in *Pinus contorta* forests. *Forest Science*, 45(3), 333-341. https://doi.org/10.1093/forestscience/45.3.333

Steele, R. (1990). *Limber Pine*. In R. M. Burns & B. H. Honkala (tech. coords.). *Silvics of North America*: *1. Conifers*. (Agriculture Handbook 654 [Vol. 2]). United States Department of Agriculture, Forest Service. https://www.srs.fs.usda.gov/pubs/misc/ag_654/volume_1/pinus/flexilis.htm

Steinberg, P. D. (2002). *Pseudotsuga menziesii* var. *glauca*. In: *Fire Effects Information System*, [Online]. United States Department of Agriculture, Forest Service, Rocky Mountain Research Station, Fire Sciences Laboratory. https://www.fs.usda.gov/database/feis/plants/tree/psemeng/all.html

Syphard, A., Scheller, R., Ward, B., Spencer, W., Strittholt, J. (2011). Simulating landscape-scale effects of fuels treatments in the Sierra Nevada, California, USA. *International Journal of Wildland Fire. 20*, 364-383. https://doi.org/10.1071/WF09125

Thornton, P. E., Thornton, M. M., Mayer, B. W., Wei, Y., Devarakonda, R., Vose, R. S., & Cook, R. B. (2016). *Daymet: Daily surface weather data on a 1-km grid for North America, version 3* [Dataset]. ORNL DAAC. https://doi.org/10.3334/ORNLDAAC/1328

Tremblay, J. A., Boulanger, Y., Cyr, D., Taylor, A. R., Price, D. T., & St-Laurent, M.-H. (2018). Harvesting interacts with climate change to affect future habitat quality of a focal species in eastern Canada's boreal forest. *PLoS One*, *13*(2). https://doi.org/10.1371/journal.pone.0191645

Uchytil, R. J. (1991a). *Abies lasiocarpa*. In *Fire Effects Information System*, [Online]. United States Department of Agriculture, Forest Service, Rocky Mountain Research Station, Fire Sciences Laboratory. https://www.fs.usda.gov/database/feis/plants/tree/abilas/all.html

Uchytil, R. J. (1991b). *Picea engelmannii*. In *Fire Effects Information System*, [Online]. United States Department of Agriculture, Forest Service, Rocky Mountain Research Station, Fire Sciences Laboratory. https://www.fs.usda.gov/database/feis/plants/tree/piceng/all.html

United States Department of Agriculture, Forest Service. (n.d.). ArcGIS Services for ~2002 basal area by species. https://apps.fs.usda.gov/fsgisx01/rest/services/RDW_FHP_TreeSpeciesMetrics

United States Department of Agriculture, Forest Service. (2020). *Design and Analysis Toolkit for Inventory and Monitoring: Welcome to DATIM!* https://apps.fs.usda.gov/datim/Default.aspx

United States Department of Agriculture, Forest Service. (2024, June 11). *Forest Inventory and Analysis*. https://research.fs.usda.gov/programs/fia

United States Department of Agriculture, Forest Service, Northern Research Station. (2019-2020). *Forest Inventory and Analysis Database* [Data set]. https://apps.fs.usda.gov/fia/datamart/datamart.html

United States Department of Agriculture, Natural Resources Conservation Service, National Geospatial Center of Excellence. (2012). *1981-2010 annual average raster precip and temp by state* [Data set]. https://nrcs.app.box.com/v/gateway/folder/22218793639

United States Department of Agriculture, Natural Resources Conservation Services, National Geospatial Center of Excellence. (2019). *National Elevation Dataset 30 meter* [Data set]. https://datagateway.nrcs.usda.gov/

United States Environmental Protection Agency, & United States Geological Survey. (2012). *National Hydrography Dataset Plus – NHDPlus Version 2.1* [Data set]. https://www.epa.gov/waterdata/nhdplus-national-hydrography-dataset-plus

United States Geological Survey. (2020). *USGS Geo Data Portal*. https://cida.usgs.gov/gdp/

Vukomanovic, J. College of Natural Resources, North Carolina State University, Raleigh, NC. Personal communication, April 14, 2020.

Weir, S. K. (2014). Colorado junipers. In *The native trees of Colorado*. Western Explorers. http://www.westernexplorers.us/ColoradoJunipers.pdf

Zeleznik, J. (n.d.). *Normal fall needle drop in conifers*. North Dakota State University (NDSU). https://web.archive.org/web/20150105115311/http://www.ag.ndsu.edu:80/horticulture/normal-fall-needle-drop-in-conifers
